# Abl depletion via autophagy mediates the beneficial effects of quercetin against Alzheimer pathology across species

**DOI:** 10.1038/s41420-023-01592-x

**Published:** 2023-10-14

**Authors:** Alfonso Schiavi, Claudia Cirotti, Lora-Sophie Gerber, Giulia Di Lauro, Silvia Maglioni, Priscila Yumi Tanaka Shibao, Sabrina Montresor, Janine Kirstein, Patrick Petzsch, Karl Köhrer, Roel P. F. Schins, Tina Wahle, Daniela Barilà, Natascia Ventura

**Affiliations:** 1 Leibniz Research Institute for Environmental Medicine (IUF), 40225 Düsseldorf, Germany; 2 Department of Biology, University of Rome “Tor Vergata”, 00133 Rome, Italy; 3 Laboratory of Cell Signaling, IRCCS-Fondazione Santa Lucia, 00179 Rome, Italy; 4 Biological and Medical Research Center (BMFZ), Medical Faculty, Heinrich-Heine-University, 40225 Duesseldorf, Germany; 5 Department of Cell Biology, University of Bremen, Bremen, Germany; 6 Leibniz Institute on Aging, Fritz Lipmann Institute, Jena, Germany; 7 Institute of Clinical Chemistry and Laboratory Diagnostic, Medical Faculty, Heinrich-Heine-University, 40225 Düsseldorf, Germany; 8 Present Address: Institute for Risk Assessment Sciences (IRAS), Faculty of Veterinary Medicine, Utrecht University, Utrecht, The Netherlands

**Keywords:** Neural ageing, Experimental models of disease

## Abstract

Alzheimer’s disease is the most common age-associated neurodegenerative disorder and the most frequent form of dementia in our society. Aging is a complex biological process concurrently shaped by genetic, dietary and environmental factors and natural compounds are emerging for their beneficial effects against age-related disorders. Besides their antioxidant activity often described in simple model organisms, the molecular mechanisms underlying the beneficial effects of different dietary compounds remain however largely unknown. In the present study, we exploit the nematode *Caenorhabditis elegans* as a widely established model for aging studies, to test the effects of different natural compounds in vivo and focused on mechanistic aspects of one of them, quercetin, using complementary systems and assays. We show that quercetin has evolutionarily conserved beneficial effects against Alzheimer’s disease (AD) pathology: it prevents Amyloid beta (Aβ)-induced detrimental effects in different *C. elegans* AD models and it reduces Aβ-secretion in mammalian cells. Mechanistically, we found that the beneficial effects of quercetin are mediated by autophagy-dependent reduced expression of Abl tyrosine kinase. In turn, autophagy is required upon Abl suppression to mediate quercetin’s protective effects against Aβ toxicity. Our data support the power of *C. elegans* as an in vivo model to investigate therapeutic options for AD.

## Introduction

Aging is a complex biological process concurrently shaped by genetic, dietary and environmental factors [1]. The aging process is characterized by progressive accumulation of damage to intracellular structures with consequent decline of different physiological functions leading to time-dependent increase in frailty and probability to die. The aging population of the industrialized world has exponentially grown in the last few decades thanks to the extension of the average human lifespan. Despite being a positive trend, this is unfortunately also associated with the increased appearance of different comorbidities and disabilities, which represent a huge economic and societal burden. Aging is therefore considered one of the most important risk factors for the development and progression of different disorders and there is an urgent need to understand its underlying molecular mechanisms to develop targeted strategies to delay or prevent the occurrence of age-associated pathologies. Alzheimer’s disease (AD) is the most common age-associated neurodegenerative disorder and the most frequent form of dementia in our society [2]. AD is ascribed to the accumulation of toxic Aβ peptide, which derives from pro-amyloidogenic proteolytic processing cleavage of the amyloid precursor protein (APP). APP is synthesized as an immature precursor protein (iAPP) and the following post-translational modification (e.g., glycosylation, phosphorylation) influence its consequent intracellular distribution. Mature APP (mAPP) is a transmembrane protein with the C-terminus located in the cytosol and its N-terminus in the lumen of different cellular vesicular compartments or in the extracellular space. mAPP can follow anti- or pro-amyloidogenic processing depending on whether it is initially cleaved by α- or β-secretase respectively, leading to the formation of soluble N-terminal (APPα and APPβ) and membrane C-terminal (APP-CTFα and APP-CTFβ) fragments. The first cleavage is then followed by an additional cleavage of APP-CTFα and APP-CTFβ by the γ-secretase, which ultimately leads to the production of either p3 or Aβ in the extracellular milieu or vesicles’ lumen, and to the APP intracellular domain (AICD) in the cytosolic compartment [3, 4]. Therefore, the amount of secreted Aβ peptide is a very sensitive readout correlating with disease pathology and it represents an important endpoint to evaluate the potential protective or detrimental properties of interventions for AD pathogenesis. AD has been primarily ascribed to accumulation of Aβ-fibrillar plaques in the brain but accumulating evidence are also revealing neuronal damage due to intracellular Aβ peptides and oligomeric deposits [5]. Thus, while in the past most therapeutic strategies were revolved towards prevention of APP pro-amyloidogenic cleavage and Aβ aggregation, additional strategies nowadays point towards shifting the oligomeric to fibrillar forms to prevent their toxic effects [6, 7].

In the last 50 years, also thanks to the exponential growth of research exploiting simple but powerful model organisms as more feasible alternatives to primate longevity studies, our knowledge of molecular mechanisms underlying the aging process has enormously improved [8, 9]. An emblematic example is represented by the nematode *Caenorhabditis elegans*, that, thanks to its short lifespan and evolutionarily conserved genome, metabolic and signaling pathways, became instrumental for aging research leading to the identification of most genes and interventions nowadays known to modulate the aging process [10, 11]. Different *C. elegans* strains overexpressing human pathogenetic Aβ peptide under muscle or neuronal specific promoters have been generated and are extensively used as alternative and powerful AD models to unravel molecular mechanisms underlying Aβ toxicity as well as for preclinical testing of potential therapeutics [12–14]. Besides classical pharmacological approaches, nutraceuticals and plant-derived natural compounds are raising interest for their potential beneficial, pro-health effects especially against chronic diseases and aging. A vast literature indicated beneficial roles for specific food components (e.g., resveratrol, curcumin, spermidine) on age-related disorders such as cancer, diabetes or cardiovascular diseases and demonstrated their pro-longevity effects in simple model organisms such as *C. elegans*. The protective effects of these natural polyphenols have been in many cases ascribed to their antioxidant activity. Moreover, induction of autophagy, a key process for the recycling of old or damaged cellular components, has been also in some cases identified as a common denominator of the protective effects of dietary interventions [15–19]. Besides their antioxidant and pro-autophagic role, little is known about more precise molecular mechanisms underlying natural compounds pro-health effects.

Here, we exploited well-established *C. elegans* models for AD to test the beneficial effects of different natural compounds in vivo and focused on mechanistic aspects of one of them, quercetin, using complementary systems and assays. We found that quercetin has evolutionarily conserved beneficial effects against AD pathology: it prevents Aβ-induced detrimental effects in the different *C. elegans* AD models and it reduces Aβ-secretion in mammalian cells. Mechanistically, an unbiased transcriptomics approach revealed phosphorylation/dephosphorylation-related processes are over-represented in quercetin treated animals. Of note, we found that reduced expression of Abl tyrosine kinase mediates the beneficial effects of quercetin and protects against Aβ-induced toxicity in *C. elegans*. Importantly, we showed that Abl expression is reduced by quercetin in cellular models of AD in an autophagy-dependent manner. Accordingly, suppression of autophagy prevented the beneficial effects of quercetin against Aβ-induced toxicity in *C. elegans* only in the presence of Abl. Moreover, we found autophagy is also required to mediate the beneficial effects of Abl suppression against Aβ toxicity. Overall, we demonstrate that quercetin has a protective activity against AD pathological features across species and suggest a positive feedback loop between Abl depletion and autophagy induction underlies its beneficial effects. Moreover, our data support the power of *C. elegans* as an in vivo model to investigate therapeutic options for Alzheimer disease.

## Results

### Quercetin promotes healthspan and protects against Aβ-induced pathology in *C. elegans*

**Fig. 1:**
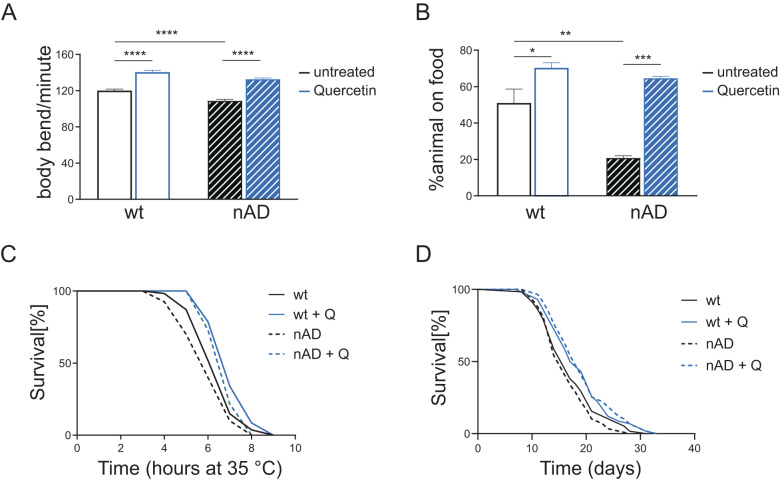
Quercetin promotes healthspan and protects against Aβ-induced pathology in *C. elegans*. **A** Body bends for minute in liquid media, of neuronal Aβ-expressing (nAD) and control (wt) worms left untreated or treated with Quercetin [100 µM], bar graph represents mean ± SEM (*N* = 2, *n* = 60), ****P < 0.0001 calculated with 2-way ANOVA (Tukey´s multiple comparisons test). **B** Percentage of 7 days old nAD and wt animals on the food 2 h after, from seeding them on the test plates, left untreated or treated with Quercetin [100 µM]. Bar graph represents mean ± SEM (*N* = 3, *n* ≥ 100), *P < 0.05, **P < 0.01, ***P < 0.001, calculated with 2-way ANOVA (Tukey´s multiple comparisons test). **C** Survival curves in response to heat shock of nAD and wt worms treated as **A**, see Table 1 for statistics. **D** Lifespan curves of wt and nAD animals treated as (**A**), see Table 2 for statistics.

Plant-derived compounds, such as polyphenols and carotenoids, are emerging as promising and very feasible dietary interventions to promote healthy aging and delay the development and progression of different age-associated disorders [18, 19]. Yet, the underlying molecular mechanisms beyond their beneficial effects are still poorly characterized. The nematode *C. elegans* offers the unique opportunity to gain insight into compounds’ mode of action in a multicellular system in vivo and it is widely used for dietary intervention studies. Moreover, there exist well-established *C. elegans* models to study the toxic effects of aggregation prone proteins, including Aβ [3, 14], and polyphenols were already shown to protect against paralysis induced by overexpression of human amyloid-beta Aβ_1-42_ under muscle specific promoter [20–22] (a strain which we will refer to as mAD in this study). Here we used a *C. elegans* strain expressing human Aβ_1-42_ under a pan neuronal promoter [23] (which we will refer to as nAD in this study) to test the protective effects of two flavonoids, quercetin and epigallocatechin gallate (EGCG), and of two carotenoids, lutein and lycopene. Compared to wild-type (wt) animals, Aβ-neuronal expression significantly reduced neuromuscular activities, such as body bends and attraction to food (Fig. 1A, B, black bars), the latter being much more dramatically affected than animal movement, likely reflecting the additive negative effects of Aβ accumulation in motor and sensory neurons. Moreover, compared to wt, nAD animals were more sensitive to stress (Fig. 1C, black lines; Table 1), but, differently from other findings [23], we did not observe differences on lifespan between the two *C. elegans* strains (Fig. 1D, black lines; Table 2). Interestingly, while compounds feeding from embryos improved motility to the same extent in both wt and nAD strains (Fig. 1A; Fig. S1A), these increased attraction to food primarily in the Aβ overexpressing animals (Fig. 1B; Fig. S1B). This may indicate a specific protective effect in certain type of neurons only in the compromised background. Of note, while quercetin significantly increased lifespan and heat-shock resistance to a similar extent in wt and nAD strains (Fig. 1C, D; Tables 1, 2), the other compounds mainly improved these parameters in the Aβ-overexpressing strain, at least in the conditions used in this work (Fig. S1C–F; Tables 1, 2). These results indicate that while all compounds display protection against Aβ overexpression (i.e., attraction to food, Fig. S1G), quercetin also promotes more general or systemic beneficial effects irrespective of genetic background and/or neuronal damage (i.e., lifespan, Fig. S1H). The beneficial effect of quercetin against different type of stressors and aging has been recognized in different model organisms [24–26], but its protection against age-associated neuropathologies has not been actively investigated and we therefore selected it for follow up studies in this work.

**Table 1:** Heat shock summary.

	Strain	Treatment	Mean Survival (h)	Standard Error	*P* vs untreated^a^	*P* vs wt	Total/Censor	*N*
Fig. 1C	GRU101 (wt)	-	6.9	0.1			52/15	3
		Quercetin [100 µM] (Q)	6.1	0.2	0.04		49/15	3
	GRU102 (nAD)	-	6.9	0.1		ns	48/12	3
		Quercetin [100 µM] (Q)	7.4	0.2	0.04	0.03	52/15	3
Fig. 3A	GRU101 (wt)	-	7.1	0.3			34/5	3
		Imatinib [1 µM] (STI)	7.9	0.3	0.03		33/6	3
	GRU102 (nAD)	-	5.7	0.3		0.01	34/2	3
		Imatinib [1 µM] (STI)	7.1	0.3	0.01	ns	32/4	3
Fig. 3B	NV48 (wt)	-	7.2	0.2			48/4	4
		*abl-1* RNAi	7.5	0.2	ns		52/4	4
	NV49 (nAD)	-	6.4	0.2		0.03	53/2	4
		*abl-1* RNAi	6.9	0.2	ns	ns	59/3	4
Fig. 3C	NV48 (wt)	-	7.1	0.3			45/9	4
	NV50 (*abl-1* KO)	-	6.3	0.3		0.01	51/7	4
	NV49 (nAD)	-	5.7	0.3		0.002	44/2	4
	NV51 (nAD;*abl-1* KO)	-	6.3	0.3		ns	43/3	4
Fig. S1D,F	GRU101 (wt)	-	7.9	0.2			50/14	3
		Lutein [100 µM]	8.8	0.1	<0.0001		58/22	3
		Lycopene [4.6 µM]	8.7	0.1	0.003		54/18	3
		EGCG [0.64 µM]	8.8	0.1	0.0007		56/20	3
	GRU102 (nAD)	-	7.1	0.2		0.003	50/14	3
		Lutein [100 µM]	8.5	0.1	<0.0001	0.001	54/18	3
		Lycopene [4.6 µM]	8.3	0.1	<0.0001	ns	53/17	3
		EGCG [0.64 µM]	8.5	0.1	<0.0001	ns	55/19	3
Fig. S2A	NV48 (wt)	Imatinib [1 µM] (STI)	7.9	0.3			33/6	3
	NV49 (nAD)	Imatinib [1 µM] (STI)	7.1	0.4	ns	ns	32/4	3
	NV51 (nAD;*abl-1* KO)	Imatinib [1 µM] (STI)	7.6	0.3		ns	35/6	3

^a^Pairwise comparisons using Log-Rank test.

**Table 2:** Lifespan summary.

	Strain	Treatment	Mean Lifespan (days)	Standard Error	*P* vs untreated^a^	*P* vs wt	Age at 100% mortality (days)	Total/Censor	*N*
Fig. 1D	GRU101 (wt)	-	16.9	0.4			31	181/7	3
		Quercetin [100 µM] (Q)	19.7	0.5	0.0001		35	180/10	3
	GRU102 (nAD)	-	16.1	0.3		ns	29	180/4	3
		Quercetin [100 µM] (Q)	19.3	0.4	<0.0001	ns	34	184/10	3
Fig. 4A-B	NV48 (wt)	-	20.5	0.5			37	200/18	3
		Quercetin [100 µM] (Q)	24.1	0.5	<0.0001		42	200/16	3
	NV50 (*abl-1* KO)	-	19.7	0.4		ns	34	200/10	3
		Quercetin [100 µM] (Q)	21.9	0.5	0.0011	ns	40	200/16	3
	NV49 (nAD)	-	18.1	0.4		0.002	32	200/18	3
		Quercetin [100 µM] (Q)	22.0	0.5	<0.0001	ns	40	200/14	3
	NV51 (nAD;*abl-1* KO)	-	22.6	0.5		0.0295	40	200/12	3
		Quercetin [100 µM] (Q)	23.3	0.5	ns	ns	40	200/11	3
FIG. S1C,E	GRU101 (wt)	-	19.1	0.5			31	140/6	3
		Lutein [100 µM]	20.6	0.5	ns		35	140/2	3
		Lycopene [4.6 µM]	20.4	0.4	ns		31	140/6	3
		EGCG [0.64 µM]	19.5	0.4	ns		31	140/5	3
	GRU102 (nAD)	-	18.8	0.4		ns	28	140/4	3
		Lutein [100 µM]	20.5	0.5	<0.0001	ns	33	140/4	3
		Lycopene [4.6 µM]	21.7	0.4	<0.0001	ns	33	140/2	3
		EGCG [0.64 µM]	21.2	0.4	0.0005	0.02	31	140/1	3

^a^Pairwise comparisons using Log-Rank test, ***P*** adjusted using the Bonferroni method.

### Quercetin treatment and Abl depletion similarly impact on phosphorylation-related processes

**Fig. 2:**
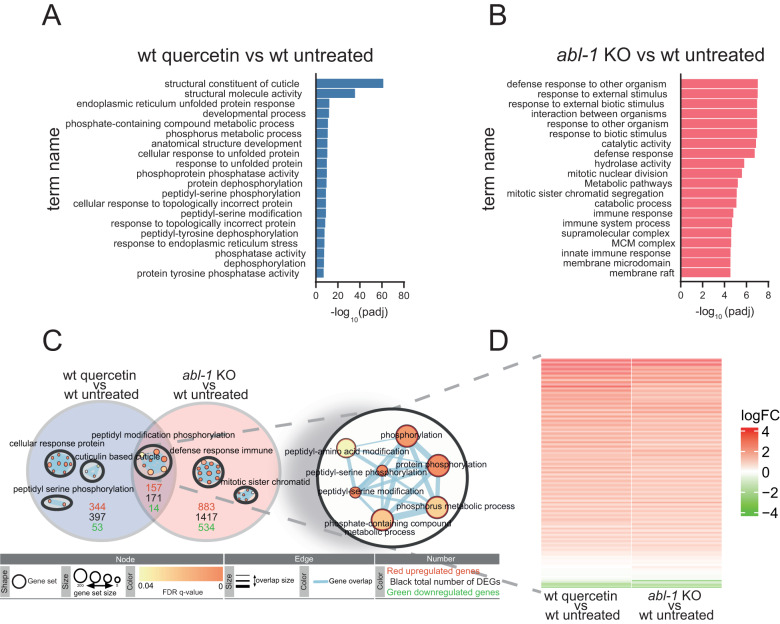
Quercetin treatment and *abl-1* depletion similarly impact on phosphorylation-related processes. Top 20 gene ontology (GO) terms found by the enrichment analysis of the differential expressed genes (DEGs) between worms left untreated or treated with 100 µM quercetin (wt quercetin vs wt untreated) **A** or *abl-1* KO strain vs wild type untreated (*abl-1* KO vs wt untreated) **B**, **C** Venn diagram build on the DEGs from RNA-Seq, the number of total DEGs, upregulated, and downregulated genes between the indicated conditions are shown in black, red and green respectively. The enrichment maps inside each circle represent the most significant functional group for each condition, the functional group inside the intersection has been enlarged to show the network in detail (circle with yellow background). **D** Heat map of log fold change of the 171 DEGs resulting from the intersection in (**C**).

Quercetin is a plant-derived flavonoid belonging to polyphenol family and it is primarily found in many types of vegetables and fruits. In search of pathways modulated by quercetin which could mediate its beneficial effects we compared the gene expression profile of untreated wt animals with that of animals treated with pro-longevity doses of quercetin. Gene ontology analysis of the 568 genes significantly modulated by quercetin, revealed that phosphorylation and dephosphorylation processes, as well as processes associated with misfolded protein responses are among the top 20 most represented terms (Fig. 2A). Loss of protein homeostasis is one of the hallmarks of the aging process [1] and phosphorylation is one of the most common post-translational modifications to regulate protein turnover and activity. Accordingly, the expression or activity of different kinases is affected during aging and in age-associated neurodegenerative disorders [27–29]. Of note, Abl tyrosine kinase was found aberrantly upregulated in AD and its inhibition was shown to provide beneficial effects in different AD models [30–32]. We thus hypothesized quercetin protection against Aβ toxicity may be mediated by Abl modulation. To address this possibility, we first analyzed the gene expression profile of an available *C. elegans abl-1* (*ok171*) mutant strain in search of a common transcriptomic signature induced by quercetin treatment and Abl depletion. The analysis of significantly modulated genes and gene ontology processes revealed a completely different pattern of gene expression in *abl-1* mutants compared to quercetin treated wild-type animals (Fig. 2A, B), which indicates a role for ABL-1 in defense and immune response against external agents. Yet, we found 171 genes (157 up and 14 down) in common between the two conditions enriched in phosphorylation-related pathways (Fig. 2C), and, for the most part, regulated in the same direction (Fig. 2D; Table 3). This analysis indicates that while quercetin treatment and Abl depletion impact on different intracellular processes, they also act through, or converge on, commonly regulated genes, suggesting quercetin may indeed promote beneficial effects via Abl suppression.

**Table 3:** LogFC 171 common genes.

Gene name	Log_FC_wt_quercetin_vs_wt_untreated	LogFC_abl1_ko_vs_wt_untreated	Gene stable ID	Gene description	Human % identity	% identity…7	Human gene name	Human gene stable ID	Paralogue gene stable ID	% identity…11	Paralog % identity
abu-1	3.95	2.42	WBGene00000024	Activated in Blocked Unfolded protein response [Source:UniProtKB/TrEMBL;Acc:Q17400]	NA	NA	NA	NA	WBGene00004098	98.1176	98.1176
abu-6	4	3.4	WBGene00000029	Activated in Blocked Unfolded protein response [Source:UniProtKB/TrEMBL;Acc:O16501]	NA	NA	NA	NA	WBGene00004099	98.7113	98.7113
abu-8	1.45	1.35	WBGene00000031	Activated in Blocked Unfolded protein response [Source:UniProtKB/TrEMBL;Acc:O16511]	NA	NA	NA	NA	WBGene00000029	84.2697	96.6495
acdh-8	1.88	1.67	WBGene00019406	Acyl CoA DeHydrogenase [Source:UniProtKB/TrEMBL;Acc:Q21243]	9.98532	16.5049	ACOX2	ENSG00000168306	WBGene00015326	28.8835	28.401
acl-13	1.28	1.28	WBGene00008581	PlsC domain-containing protein [Source:UniProtKB/TrEMBL;Acc:Q19221]	18.1081	18.4573	LPGAT1	ENSG00000123684	WBGene00015295	33.6088	31.202
acp-3	3.1	3.1	WBGene00008801	ACid Phosphatase family [Source:UniProtKB/TrEMBL;Acc:Q19460]	16.7464	16.4706	ACP3	ENSG00000014257	WBGene00017427	14.1176	14.4231
alg-3	1.8	1.62	WBGene00011910	Argonaute (Plant)-Like protein [Source:UniProtKB/TrEMBL;Acc:G5ED77]	NA	NA	NA	NA	WBGene00007297	15.2657	16.7373
B0207.7	4.52	4.22	WBGene00015030	Protein kinase domain-containing protein [Source:UniProtKB/TrEMBL;Acc:O01429]	NA	NA	NA	NA	WBGene00002203	16.8901	12.6761
B0379.7	1.61	1.61	WBGene00007159	NA	NA	NA	NA	NA	WBGene00015024	54.7677	49.2308
btb-2	3.17	3.01	WBGene00020802	BTB domain-containing protein [Source:UniProtKB/TrEMBL;Acc:Q8IFY9]	NA	NA	NA	NA	WBGene00018200	34.0278	30.5296
C01G10.14	3.11	3.33	WBGene00007239	Major sperm protein [Source:UniProtKB/TrEMBL;Acc:Q93173]	NA	NA	NA	NA	WBGene00010091	25.3333	35.514
C01G5.4	2.45	3.19	WBGene00015306	WSN domain-containing protein [Source:UniProtKB/TrEMBL;Acc:Q17566]	NA	NA	NA	NA	WBGene00015630	40.2224	41.8112
C02F5.5	3.04	2.02	WBGene00015348	NA	NA	NA	NA	NA	WBGene00021398	51.4451	55.9748
C04F12.7	2.37	1.94	WBGene00007301	NA	NA	NA	NA	NA	WBGene00018314	96.5347	96.5347
C08F8.6	1.84	1.64	WBGene00007448	Protein kinase domain-containing protein [Source:UniProtKB/TrEMBL;Acc:Q17825]	NA	NA	NA	NA	WBGene00002203	18.9474	14.4869
C09B9.4	2.87	2.24	WBGene00015629	Protein kinase domain-containing protein [Source:UniProtKB/TrEMBL;Acc:Q17853]	NA	NA	NA	NA	WBGene00002203	18.3844	13.2797
C09H10.9	1.98	1.72	WBGene00007503	NA	NA	NA	NA	NA	WBGene00015765	19.346	20.5202
C10G11.8	2.12	1.72	WBGene00015688	AAA domain-containing protein [Source:UniProtKB/TrEMBL;Acc:P91025]	NA	NA	NA	NA	WBGene00004502	54.3379	53.7246
C14C10.1	2.46	2.16	WBGene00007584	NA	NA	NA	NA	NA	WBGene00011493	64.9701	55.2163
C15C6.2	2.47	2.08	WBGene00007601	NA	NA	NA	NA	NA	WBGene00011176	31.1419	30.1003
C18A3.7	3.5	3.13	WBGene00015944	NA	NA	NA	NA	NA	NA	NA	NA
C24H11.1	−4.38	−4.53	WBGene00007699	Serine/threonine-protein phosphatase [Source:UniProtKB/TrEMBL;Acc:Q9U3P4]	NA	NA	NA	NA	WBGene00015661	31.7708	36.6366
C25D7.16	2.07	1.92	WBGene00050940	NA	NA	NA	NA	NA	NA	NA	NA
C27D6.11	2.09	2.28	WBGene00044388	Protein kinase domain-containing protein [Source:UniProtKB/TrEMBL;Acc:Q4U220]	11.054	25.9819	BRSK1	ENSG00000160469	WBGene00021012	29.6073	13.881
C28D4.5	2.98	2.3	WBGene00007793	DUF1248 domain-containing protein [Source:UniProtKB/TrEMBL;Acc:O17609]	NA	NA	NA	NA	WBGene00009184	1.69972	7.40741
C32D5.4	5.69	4.41	WBGene00016312	NA	NA	NA	NA	NA	NA	NA	NA
C32E8.4	2.04	1.81	WBGene00016322	NA	NA	NA	NA	NA	WBGene00045209	91.6667	90.1639
C33F10.1	1.61	1.59	WBGene00016351	NA	NA	NA	NA	NA	WBGene00016357	89.759	81.8681
C35A11.2	2.53	2.37	WBGene00016429	NA	NA	NA	NA	NA	NA	NA	NA
C38C3.3	3.84	3.16	WBGene00016512	NA	NA	NA	NA	NA	WBGene00044177	84.5395	85.3821
C39H7.1	2.06	1.81	WBGene00016541	Protein kinase domain-containing protein [Source:UniProtKB/TrEMBL;Acc:Q18553]	NA	NA	NA	NA	WBGene00012637	98.0519	98.0519
C43 G2.3	2.11	2.4	WBGene00016612	NA	NA	NA	NA	NA	WBGene00045355	29.0476	26.9911
C45G9.9	2.05	1.68	WBGene00016680	NA	NA	NA	NA	NA	WBGene00016675	96.9388	97.6027
C46A5.1	1.2	1.6	WBGene00016698	NA	NA	NA	NA	NA	WBGene00021702	14.4869	19.9446
C48B4.11	0.13	0.11	WBGene00008174	NA	NA	NA	NA	NA	NA	NA	NA
C50F2.5	2.43	2.53	WBGene00016839	Tyrosine-protein phosphatase domain-containing protein [Source:UniProtKB/TrEMBL;Acc:P91179]	NA	NA	NA	NA	WBGene00021702	12.7717	13.0194
C53D6.10	2.84	2.72	WBGene00023424	NA	NA	NA	NA	NA	NA	NA	NA
C54G4.2	2.69	2.76	WBGene00008312	NA	NA	NA	NA	NA	WBGene00007489	30.9693	33.1646
col-120	3.1	2.03	WBGene00000694	Col_cuticle_N domain-containing protein [Source:UniProtKB/TrEMBL;Acc:Q9XWR2]	NA	NA	NA	NA	WBGene00000636	27.7955	28.8079
col-13	1.93	1.33	WBGene00000602	Cuticle collagen 13 [Source:UniProtKB/Swiss-Prot;Acc:P20631]	NA	NA	NA	NA	WBGene00000636	32.2785	33.7748
col-146	3.29	2.01	WBGene00000719	Col_cuticle_N domain-containing protein [Source:UniProtKB/TrEMBL;Acc:Q22260]	NA	NA	NA	NA	WBGene00000636	29.6552	28.4768
col-156	3.67	2.25	WBGene00000729	Col_cuticle_N domain-containing protein [Source:UniProtKB/TrEMBL;Acc:Q20927]	NA	NA	NA	NA	WBGene00000636	32.2034	31.457
col-77	2.57	1.72	WBGene00000653	Col_cuticle_N domain-containing protein [Source:UniProtKB/TrEMBL;Acc:Q21562]	NA	NA	NA	NA	WBGene00000636	29.2763	29.4702
col-91	3.25	2.46	WBGene00000666	Putative cuticle collagen 91 [Source:UniProtKB/Swiss-Prot;Acc:P34391]	NA	NA	NA	NA	WBGene00000636	30.9353	28.4768
col-92	0.7	0.8	WBGene00000667	Col_cuticle_N domain-containing protein [Source:UniProtKB/TrEMBL;Acc:Q9XVG3]	NA	NA	NA	NA	WBGene00000636	26.3158	26.4901
comp-1	3.02	2.38	WBGene00018158	Protein kinase domain-containing protein [Source:UniProtKB/TrEMBL;Acc:O01765]	NA	NA	NA	NA	NA	NA	NA
cpb-2	1.95	1.7	WBGene00000771	Cytoplasmic polyadenylation element-binding protein 2 [Source:UniProtKB/Swiss-Prot;Acc:Q18317]	12.6692	22.9825	CPEB2	ENSG00000137449	WBGene00000772	17.5439	13.4228
cyc-2.2	1.89	1.49	WBGene00013854	Probable cytochrome c 2.2 [Source:UniProtKB/Swiss-Prot;Acc:Q23240]	54.2857	46.3415	CYCS	ENSG00000172115	WBGene00017121	70.7317	78.3784
cyp-35A3	−0.64	-5.59	WBGene00019565	CYtochrome P450 family [Source:UniProtKB/TrEMBL;Acc:Q9N5I1]	27.7551	27.5304	CYP2C19	ENSG00000165841	WBGene00015709	27.7328	27.8455
D1081.5	2.41	2.72	WBGene00008383	NA	NA	NA	NA	NA	WBGene00018841	62.1818	61.2903
D1086.17	2.05	1.59	WBGene00045355	NA	NA	NA	NA	NA	WBGene00016612	26.9911	29.0476
eas-1	0.12	0.25	WBGene00018270	Probable Golgi transport protein 1 [Source:UniProtKB/Swiss-Prot;Acc:Q20263]	NA	NA	NA	NA	NA	NA	NA
egl-19	0.18	0.31	WBGene00001187	Voltage-dependent L-type calcium channel subunit alpha [Source:UniProtKB/TrEMBL;Acc:G5EG02]	49.4183	52.0511	CACNA1F	ENSG00000102001	WBGene00003558	16.9952	17.8711
F07A5.2	1.26	1.16	WBGene00008541	NA	NA	NA	NA	NA	NA	NA	NA
F08H9.2	1.76	1.66	WBGene00008590	NA	NA	NA	NA	NA	NA	NA	NA
F09C12.8	1.93	1.72	WBGene00017279	NA	11.3464	24.5098	UBASH3A	ENSG00000160185	WBGene00010082	23.5294	22.9299
F10C1.23	3.49	3.03	WBGene00271819	NA	NA	NA	NA	NA	WBGene00017325	95.5556	95.5556
F10G8.1	3.06	2.75	WBGene00008661	Tyrosine-protein phosphatase [Source:UniProtKB/TrEMBL;Acc:I2HAD7]	NA	NA	NA	NA	WBGene00021702	30.0578	28.8089
F17E9.5	2.33	1.8	WBGene00017542	NA	NA	NA	NA	NA	WBGene00021398	80.2469	81.761
F21H7.5	1.9	1.84	WBGene00009031	Major sperm protein [Source:UniProtKB/TrEMBL;Acc:O45386]	NA	NA	NA	NA	WBGene00010091	11.1111	31.7757
F25H2.7	0.23	0.27	WBGene00009121	NA	NA	NA	NA	NA	WBGene00012049	25.8152	26.3889
F26B1.8	2.71	2.17	WBGene00194703	NA	NA	NA	NA	NA	WBGene00020350	84.9057	88.8158
F26F4.2	1.56	1.7	WBGene00005012	NA	NA	NA	NA	NA	WBGene00009501	74.0331	75.7062
F32H2.7	1.76	1.55	WBGene00009344	NA	NA	NA	NA	NA	NA	NA	NA
F35H8.4	3.76	3.49	WBGene00009449	NA	NA	NA	NA	NA	NA	NA	NA
F36H1.3	3.33	3.15	WBGene00009492	Tyrosine-protein phosphatase [Source:UniProtKB/TrEMBL;Acc:Q20108]	NA	NA	NA	NA	WBGene00021702	17.8571	26.3158
F36H12.9	2.71	2.28	WBGene00018123	Protein kinase domain-containing protein [Source:UniProtKB/TrEMBL;Acc:O76711]	NA	NA	NA	NA	WBGene00002203	20.7895	15.8954
F37A4.4	1.54	1.85	WBGene00018134	Ankyrin repeat-containing protein F37A4.4 [Source:UniProtKB/Swiss-Prot;Acc:P41882]	NA	NA	NA	NA	WBGene00015988	31.9003	30.9425
F37A4.5	1.2	1.32	WBGene00018135	NA	15.1899	15.047	BRCC3	ENSG00000185515	WBGene00000817	25.3918	22.0109
F41H10.1	1.95	1.63	WBGene00018314	NA	NA	NA	NA	NA	WBGene00007301	96.5347	96.5347
F44B9.10	1.24	1.37	WBGene00018411	NA	11.7949	19.6581	PLA2G12B	ENSG00000138308	WBGene00016288	30.7692	11.4286
F44G3.7	3.07	2.83	WBGene00009708	NA	NA	NA	NA	NA	WBGene00008854	39.4326	33.7789
F47H4.2	0.99	1.23	WBGene00009835	NA	NA	NA	NA	NA	WBGene00011212	24.3217	15.2398
F52F12.5	1.61	1.87	WBGene00009938	NA	NA	NA	NA	NA	NA	NA	NA
F52H3.6	1.7	1.66	WBGene00009948	Serine/threonine-protein phosphatase [Source:UniProtKB/TrEMBL;Acc:Q27501]	NA	NA	NA	NA	WBGene00021113	49.848	53.7705
F53C3.1	1.88	1.9	WBGene00018745	Protein kinase domain-containing protein [Source:UniProtKB/TrEMBL;Acc:Q9TXU0]	NA	NA	NA	NA	WBGene00002203	23.1707	15.2918
F54D1.1	1.81	2.3	WBGene00010046	NA	12.894	16.187	KHDRBS2	ENSG00000112232	WBGene00013325	16.9065	12.3684
F55A12.6	0.84	0.97	WBGene00018865	NA	NA	NA	NA	NA	WBGene00195179	28.5047	36.9697
F55B11.2	−0.12	-0.12	WBGene00010084	NA	NA	NA	NA	NA	NA	NA	NA
F55H12.5	1.56	1.45	WBGene00010136	Tyrosine-protein phosphatase domain-containing protein [Source:UniProtKB/TrEMBL;Acc:Q8I4I0]	NA	NA	NA	NA	WBGene00021702	13.5889	10.8033
F58F12.2	4.06	4.18	WBGene00019062	NA	NA	NA	NA	NA	NA	NA	NA
F59B2.8	0.92	0.92	WBGene00010310	NA	NA	NA	NA	NA	WBGene00010311	43.2184	44.2353
fipr-7	1.27	1.5	WBGene00007543	FIP (Fungus-Induced Protein) Related [Source:UniProtKB/TrEMBL;Acc:Q7YTS1]	NA	NA	NA	NA	WBGene00044175	93.1507	93.1507
frk-1	2.28	2.18	WBGene00001487	Fer-related kinase 1 [Source:UniProtKB/Swiss-Prot;Acc:Q22146]	NA	NA	NA	NA	WBGene00022634	25.8974	25.5696
frpr-12	1.28	1.3	WBGene00019445	G_PROTEIN_RECEP_F1_2 domain-containing protein [Source:UniProtKB/TrEMBL;Acc:Q9GYH3]	NA	NA	NA	NA	WBGene00007951	8.50515	6.77618
gipc-1	1.83	1.67	WBGene00016440	PDZ domain-containing protein [Source:UniProtKB/TrEMBL;Acc:Q18488]	31.746	28.0112	GIPC2	ENSG00000137960	WBGene00009681	79.2717	79.2717
grd-3	-0.12	0.2	WBGene00001692	Ground-like domain-containing protein [Source:UniProtKB/TrEMBL;Acc:Q9TYW7]	4.329	11.0497	SHH	ENSG00000164690	WBGene00006955	9.94475	3.25497
grd-6	2.75	2.35	WBGene00001695	Ground-like domain-containing protein [Source:UniProtKB/TrEMBL;Acc:A0A3P6NID2]	3.67965	2.76873	SHH	ENSG00000164690	WBGene00006955	4.07166	4.5208
gsp-3	1.69	1.47	WBGene00021113	Serine/threonine-protein phosphatase PP1-gamma [Source:UniProtKB/Swiss-Prot;Acc:O02658]	NA	NA	NA	NA	WBGene00020187	98.0328	98.0328
H20J04.1	3.49	2.29	WBGene00019216	WSN domain-containing protein [Source:UniProtKB/TrEMBL;Acc:Q9N5L7]	NA	NA	NA	NA	NA	NA	NA
his-27	2.01	1.73	WBGene00001901	Histone H3 [Source:UniProtKB/Swiss-Prot;Acc:P08898]	94.1176	94.1176	H3-4	ENSG00000168148	WBGene00010036	42.6471	22.2222
ipla-5	4.21	4.57	WBGene00019229	Intracelllar PhosphoLipase A family [Source:UniProtKB/TrEMBL;Acc:Q9N5L3]	NA	NA	NA	NA	WBGene00017026	30.5835	27.289
K01D12.15	2.24	2.18	WBGene00010474	NA	NA	NA	NA	NA	WBGene00010466	100	100
K02E11.10	3.91	2.76	WBGene00044109	NA	NA	NA	NA	NA	NA	NA	NA
K06A5.2	1.68	1.65	WBGene00019430	NA	NA	NA	NA	NA	WBGene00019024	16.8675	18.7919
K06H7.8	2.35	2.3	WBGene00019459	Putative serine/threonine-protein kinase K06H7.1 [Source:UniProtKB/Swiss-Prot;Acc:P34516]	NA	NA	NA	NA	WBGene00002203	21.0983	14.6881
K08A2.2	1.29	1.25	WBGene00019512	DUF1248 domain-containing protein [Source:UniProtKB/TrEMBL;Acc:Q9N5J3]	NA	NA	NA	NA	WBGene00009184	1.7192	7.40741
K08C9.1	3.68	3.1	WBGene00010650	NA	NA	NA	NA	NA	WBGene00010651	32.5	33.1915
K09E4.1	1.11	1.4	WBGene00010719	NA	NA	NA	NA	NA	WBGene00002203	12.8065	9.45674
K11D12.13	−0.17	-0.24	WBGene00044535	BPTI/Kunitz inhibitor domain-containing protein [Source:UniProtKB/TrEMBL;Acc:Q4R127]	2.98507	5.31915	HOXA1	ENSG00000105991	WBGene00011069	6.38298	4.31655
ltd-1	0.49	0.59	WBGene00003089	TGc domain-containing protein [Source:UniProtKB/TrEMBL;Acc:A0A131MD24]	6.58579	15.7676	NRAP	ENSG00000197893	WBGene00018367	10.2351	22.0896
M05D6.1	2.89	2.16	WBGene00010874	Protein kinase domain-containing protein [Source:UniProtKB/TrEMBL;Acc:Q21521]	NA	NA	NA	NA	WBGene00002203	18.5596	13.4809
mpst-4	2.48	2.64	WBGene00017387	Putative thiosulfate sulfurtransferase mpst-4 [Source:UniProtKB/Swiss-Prot;Acc:P91247]	20.202	18.5759	TST	ENSG00000128311	WBGene00022006	64.3963	69.3333
mpz-4	1.98	2.28	WBGene00019165	PDZ domain-containing protein [Source:UniProtKB/TrEMBL;Acc:Q9TXV1]	NA	NA	NA	NA	WBGene00016843	19.9005	25.5591
msp-142	2.39	1.7	WBGene00003469	Major sperm protein 19/31/40/45/50/51/53/59/61/65/81/113/142 [Source:UniProtKB/Swiss-Prot;Acc:P53017]	NA	NA	NA	NA	WBGene00003452	100	100
msp-3	2.46	1.84	WBGene00003424	Major sperm protein 3 [Source:UniProtKB/Swiss-Prot;Acc:Q19832]	NA	NA	NA	NA	WBGene00003434	97.6378	97.6378
msp-51	2.04	1.58	WBGene00003444	Major sperm protein 19/31/40/45/50/51/53/59/61/65/81/113/142 [Source:UniProtKB/Swiss-Prot;Acc:P53017]	NA	NA	NA	NA	WBGene00003448	99.2126	99.2126
msp-57	2.43	2.03	WBGene00003450	Major sperm protein 55/57 [Source:UniProtKB/Swiss-Prot;Acc:Q17856]	NA	NA	NA	NA	WBGene00003448	100	100
msp-59	2.03	1.56	WBGene00003452	Major sperm protein 19/31/40/45/50/51/53/59/61/65/81/113/142 [Source:UniProtKB/Swiss-Prot;Acc:P53017]	NA	NA	NA	NA	WBGene00003429	100	100
msp-77	1.98	1.75	WBGene00003464	Major sperm protein 77/79 [Source:UniProtKB/Swiss-Prot;Acc:Q9TVW5]	NA	NA	NA	NA	WBGene00003466	100	100
msrp-2	1.82	1.48	WBGene00022751	MS Related Protein [Source:UniProtKB/TrEMBL;Acc:O44898]	NA	NA	NA	NA	NA	NA	NA
msrp-4	4.71	4.68	WBGene00018500	MS Related Protein [Source:UniProtKB/TrEMBL;Acc:Q9TXY4]	NA	NA	NA	NA	WBGene00018497	85.5769	83.9623
nep-23	4.15	4.23	WBGene00013785	NEPrilysin metallopeptidase family [Source:UniProtKB/TrEMBL;Acc:Q9U2T1]	14.4516	15.6863	ECEL1	ENSG00000171551	WBGene00013926	19.0476	18.0371
nep-6	2.06	2.22	WBGene00017550	NEPrilysin metallopeptidase family [Source:UniProtKB/TrEMBL;Acc:O16795]	16.5161	17.6309	ECEL1	ENSG00000171551	WBGene00013926	20.3857	19.6286
nhr-206	1.13	1	WBGene00011097	Nuclear Hormone Receptor family [Source:UniProtKB/TrEMBL;Acc:Q21803]	16.1836	16.3415	NR2F2	ENSG00000185551	WBGene00003690	14.3902	13.5011
nspd-3	2.8	2.23	WBGene00016058	Nematode Specific Peptide family, group D [Source:UniProtKB/TrEMBL;Acc:G5EG23]	NA	NA	NA	NA	WBGene00043147	100	100
nspe-1	−0.4	−0.38	WBGene00012591	Nematode Specific Peptide family, group E [Source:UniProtKB/TrEMBL;Acc:Q9NAJ8]	NA	NA	NA	NA	WBGene00012604	97.1831	97.1831
pals-4	−1.66	−1.79	WBGene00007658	Protein containing ALS2cr12 (ALS2CR12) signature [Source:UniProtKB/TrEMBL;Acc:O45256]	NA	NA	NA	NA	WBGene00044237	45.584	54.4218
phg-1	1.42	1.16	WBGene00004017	Growth arrest-specific protein 1 homolog [Source:UniProtKB/Swiss-Prot;Acc:Q09553]	NA	NA	NA	NA	NA	NA	NA
pqn-54	1.37	1.19	WBGene00004139	Prion-like-(Q/N-rich)-domain-bearing protein [Source:UniProtKB/TrEMBL;Acc:O44606]	NA	NA	NA	NA	NA	NA	NA
R02F11.1	1.06	1.25	WBGene00019839	NA	NA	NA	NA	NA	WBGene00018492	26.7045	26.4045
R08A2.2	2.97	2.69	WBGene00011133	Serine/threonine-protein phosphatase [Source:UniProtKB/TrEMBL;Acc:Q9U395]	NA	NA	NA	NA	WBGene00015661	30.9973	34.5345
R09E10.6	2.18	1.84	WBGene00011176	NA	NA	NA	NA	NA	WBGene00007601	30.1003	31.1419
R10H10.3	0.18	0.27	WBGene00011222	NA	NA	NA	NA	NA	WBGene00008879	7.96703	4.22741
R12E2.7	1.92	1.83	WBGene00020033	NA	NA	NA	NA	NA	NA	NA	NA
smz-1	0.93	1.22	WBGene00007733	Sperm Meiosis PDZ domain containing proteins [Source:UniProtKB/TrEMBL;Acc:Q18167]	NA	NA	NA	NA	WBGene00020661	98.9051	98.9051
spch-3	2.47	1.81	WBGene00020840	SPerm CHromatin enriched [Source:UniProtKB/TrEMBL;Acc:P91497]	NA	NA	NA	NA	WBGene00015689	99.0148	99.0148
spe-46	1.15	1.4	WBGene00012296	NA	NA	NA	NA	NA	NA	NA	NA
sptl-2	−0.17	−0.17	WBGene00018398	Serine palmitoyltransferase 2 [Source:UniProtKB/Swiss-Prot;Acc:Q20375]	15.7812	17.2355	ALAS1	ENSG00000023330	WBGene00011932	41.2969	46.4491
ssp-16	3.74	2.68	WBGene00006044	Sperm-specific class P protein 16 [Source:UniProtKB/Swiss-Prot;Acc:P91499]	NA	NA	NA	NA	WBGene00010091	44.9541	45.7944
ssp-31	2.21	2.24	WBGene00006048	Sperm-specific class P protein 31 [Source:UniProtKB/Swiss-Prot;Acc:Q9XXL3]	NA	NA	NA	NA	WBGene00015696	27.1028	16.3842
ssp-9	2.34	1.64	WBGene00006038	Sperm-specific class P protein 9/11 [Source:UniProtKB/Swiss-Prot;Acc:Q23058]	NA	NA	NA	NA	WBGene00010091	44.9541	45.7944
ssq-1	1.24	1.11	WBGene00006050	Sperm-Specific family, class Q [Source:UniProtKB/TrEMBL;Acc:Q21294]	NA	NA	NA	NA	WBGene00006051	89.3103	63.9506
ssq-4	2.12	1.54	WBGene00006053	Sperm-Specific family, class Q [Source:UniProtKB/TrEMBL;Acc:Q23062]	NA	NA	NA	NA	WBGene00006052	98.6595	99.1914
sss-1	2.18	1.82	WBGene00006056	Sperm-Specific family, class S [Source:UniProtKB/TrEMBL;Acc:Q9XVP7]	NA	NA	NA	NA	WBGene00017851	37.4101	38.6617
T02E1.7	4.02	3.03	WBGene00011379	NA	NA	NA	NA	NA	WBGene00004787	9.66543	8.04954
T05C12.4	1.21	1.12	WBGene00011468	NA	NA	NA	NA	NA	NA	NA	NA
T06E4.12	4.5	3.5	WBGene00044011	NA	NA	NA	NA	NA	WBGene00077691	68.9189	69.863
T08B2.12	2.2	2.36	WBGene00020350	NA	NA	NA	NA	NA	WBGene00016752	99.3421	99.3421
T08B6.4	1.74	1.56	WBGene00020353	NA	NA	NA	NA	NA	WBGene00010241	39.3027	40.5892
T16G12.7	2.06	1.92	WBGene00011808	Serine/threonine-protein phosphatase [Source:UniProtKB/TrEMBL;Acc:K8FE09]	NA	NA	NA	NA	WBGene00015661	52.0376	49.8498
T23F11.2	2.48	2.11	WBGene00011954	NA	NA	NA	NA	NA	WBGene00008912	96.4427	82.4324
T27E7.1	2.99	2.22	WBGene00012087	NA	NA	NA	NA	NA	WBGene00013138	34.0708	21.4485
T28F3.5	0.53	0.62	WBGene00012131	Acetyl-CoA carboxylase [Source:UniProtKB/TrEMBL;Acc:C1P655]	22.3759	34.1191	ACACB	ENSG00000076555	WBGene00017864	10.794	24.0331
ubxn-5	1.97	1.71	WBGene00011336	UBX domain-containing protein 5 [Source:UniProtKB/Swiss-Prot;Acc:Q7YWU9]	NA	NA	NA	NA	NA	NA	NA
ugt-23	0.18	0.18	WBGene00007650	UDP-glucuronosyltransferase [Source:UniProtKB/TrEMBL;Acc:Q0G821]	NA	NA	NA	NA	WBGene00008583	17.7358	18.4676
ugt-4	0.41	0.68	WBGene00013905	UDP-glucuronosyltransferase [Source:UniProtKB/TrEMBL;Acc:Q23335]	NA	NA	NA	NA	WBGene00008583	15.8879	16.6994
W02D9.6	−0.27	−0.29	WBGene00012212	NA	NA	NA	NA	NA	NA	NA	NA
W02D9.7	−0.16	−0.12	WBGene00012213	NA	NA	NA	NA	NA	NA	NA	NA
W03D8.9	1.97	1.63	WBGene00020990	NA	NA	NA	NA	NA	WBGene00012547	84.326	84.0625
W03F11.4	2.84	2.09	WBGene00021007	Protein-tyrosine-phosphatase [Source:UniProtKB/TrEMBL;Acc:O01777]	NA	NA	NA	NA	WBGene00020116	15.0255	16.6397
Y113G7C.1	2.6	2.37	WBGene00013771	Protein-tyrosine-phosphatase [Source:UniProtKB/TrEMBL;Acc:Q9XWA6]	NA	NA	NA	NA	WBGene00021702	4.35074	18.0055
Y17D7B.3	−3.13	−3.11	WBGene00012451	NA	NA	NA	NA	NA	NA	NA	NA
Y17G9B.11	−2.68	−2.68	WBGene00194835	NA	NA	NA	NA	NA	NA	NA	NA
Y38E10A.17	1.61	1.46	WBGene00012595	NA	NA	NA	NA	NA	NA	NA	NA
Y43F8A.2	2.4	1.78	WBGene00012809	NA	NA	NA	NA	NA	WBGene00017955	28.0877	29.8729
Y43F8C.5	2.92	2.84	WBGene00012827	NA	NA	NA	NA	NA	NA	NA	NA
Y47D7A.15	4.9	2.75	WBGene00021627	NA	NA	NA	NA	NA	NA	NA	NA
Y51B9A.5	2.76	2.8	WBGene00013087	NA	NA	NA	NA	NA	WBGene00009459	44.4444	43.0108
Y67D8B.5	1.84	1.84	WBGene00022064	NA	NA	NA	NA	NA	NA	NA	NA
Y71F9AL.11	−3.11	−1.89	WBGene00022116	NA	NA	NA	NA	NA	NA	NA	NA
ZC477.7	2.25	1.89	WBGene00022621	NA	NA	NA	NA	NA	WBGene00020905	97.4227	97.4227
ZC513.14	3.46	2.18	WBGene00194928	DUF19 domain-containing protein [Source:UniProtKB/TrEMBL;Acc:F1LIM2]	NA	NA	NA	NA	NA	NA	NA
zip-12	−0.19	0.28	WBGene00013560	BZIP transcription factor family [Source:UniProtKB/TrEMBL;Acc:Q9XW80]	NA	NA	NA	NA	NA	NA	NA
ZK1225.4	2.39	2.11	WBGene00014238	NA	NA	NA	NA	NA	WBGene00014239	57.0328	57.9256
ZK1307.4	1.94	1.69	WBGene00014247	NA	NA	NA	NA	NA	WBGene00015696	16.5217	10.7345
ZK512.8	1.79	1.4	WBGene00013987	NA	NA	NA	NA	NA	WBGene00013886	77.193	78.5714
ZK550.5	1.67	1.51	WBGene00013999	NA	42.0118	43.2927	PHYH	ENSG00000107537	WBGene00044362	17.0732	45.9016
ZK858.2	1.77	1.65	WBGene00014116	NA	NA	NA	NA	NA	NA	NA	NA
ZK938.1	1.94	1.68	WBGene00014158	Serine/threonine-protein phosphatase [Source:UniProtKB/TrEMBL;Acc:G5ECL6]	NA	NA	NA	NA	WBGene00015661	49.8471	48.9489
ZK945.7	1.88	1.64	WBGene00014169	NA	NA	NA	NA	NA	NA	NA	NA

%Genome project: caenorhabditis_elegans_prjna13758.

% expression combined with result from https://parasite.wormbase.org/.

% BioMart version 0.7.

%imput query common 171 genes list.

### Abl suppression mediates the beneficial effects of quercetin against Aβ-induced pathology

**Fig. 3:**
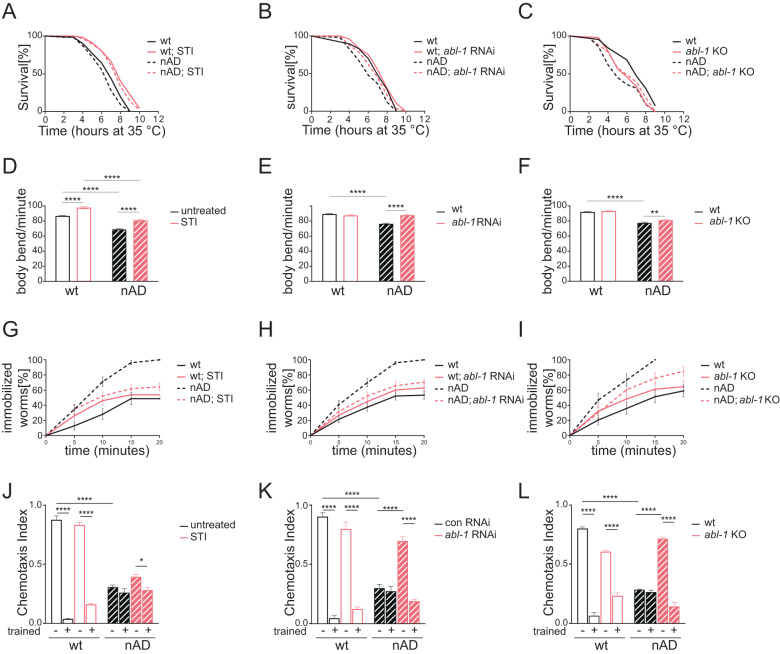
Suppression of ABL-1 tyrosine kinase protects against Aβ-induced pathology in *C. elegans*. Survival curves in response to heat shock of neuronal Aβ-expressing (nAD) and control (wt) worms, left untreated or treated with Imatinib [1 µM] (STI) (**A**), fed OP50(xu363) bacteria transformed with either empty-vector or vector-expressing dsRNA against *abl-1* (*abl-1* RNAi) (**B**), crossed with *abl-1* Knockout (*abl-1* KO) (**C**) see Table 1 for statistics. Body bends for minute in liquid media, of neuronal Aβ-expressing (nAD) and control (wt) worms, left untreated or treated with Imatinib [1 µM] (STI) (**D**), fed OP50(xu363) bacteria transformed with either empty-vector or vector-expressing dsRNA against *abl-1* (*abl-1* RNAi) (**E**), crossed with abl-1 KO (**F**). Bar graphs represent mean ± SEM (*N* = 3, *n* = 30). ****P < 0.0001, ** *P* < 0.01 calculated with 2-way ANOVA (Tukey’s multiple comparisons test). **G-I** Percentage of immobilized worms, of neuronal Aβ-expressing (nAD) and control (wt) worms, left untreated or treated with Imatinib [1 µM] (STI) (**G**), fed OP50(xu363) bacteria transformed with either empty-vector or vector-expressing dsRNA against *abl-1* (*abl-1* RNAi) (**H**), crossed with abl-1 KO (**I**). Immobilization was induced using Serotonin [10 mM] in S-basal, see Table 4 for statistics. Chemotaxis index of neuronal Aβ-expressing (nAD) and control (wt) worms, left untreated or treated with Imatinib [1 µM] (STI) (**J**), fed OP50(xu363) bacteria transformed with either empty-vector or vector-expressing dsRNA against *abl-1* (*abl-1* RNAi) (**K**), crossed with abl-1 KO (**L**). Prior to proceeding with the chemotaxis assay, worms were starved for 2 h either with (trained) or without (−) Benzaldehyde [1%]. Bar graphs represent mean ± SEM (*N* = 3, *n* ≥ 41) ****P < 0.0001 calculated with 2-way ANOVA (Tukey’s multiple comparisons test).

We then addressed whether Abl suppression could indeed have, similar to mice [30], a protective role in *C. elegans* AD models. As described above, overexpression of neuronal Aβ decreases animals’ resistance to heat shock (Fig. 3A–C, survival time at 35 °C; Table 1) and locomotion activity (Fig. 3D–F, number of body bends/min). Remarkably, we found that pharmacological inhibition of the *C. elegans* Abl homolog, ABL-1, with imatinib (STI) or its genetic depletion via silencing (*abl-1* RNAi) or knock-out (*abl-1* KO), significantly improve survival upon heat-shock (Fig. 3A–C; Table 1) and locomotion activity (Fig. 3D–F) in the nAD strain. Moreover, according to a previous study (which used a strain expressing Aβ under a different neuronal promoter [33]), we showed here that neuronal Aβ overexpression sensitizes animals to serotonin-induced paralysis and, most importantly, this defect is also rescued by pharmacological or genetic suppression of *abl-1* (Fig. 3G–I; Table 4). It is worth noting that *abl-1* suppression had negligible effects in the control strain not expressing Aβ, on stress resistance, locomotion or serotonin-induced paralysis (Fig. 3A–I; Tables 1, 4). Importantly, we found that genetic ablation of *abl-1* reduces neuronal Aβ aggregation with aging. Strikingly, this occurred in the IL2 neurons, a subset of head neurons, which mark the onset of Aβ fibril formation, spreading and pathology [34] (Fig. S2A–E).

**Table 4:** Serotonin assay summary.

	Strain	Treatment	Mean Immobilizing time (min)	Standard Error	*P* vs untreated^a^	*P* vs wt	Total/Censor	*N*
Fig. 3G	GRU101 (wt)	-	15.6	0.9			39/20	4
		Imatinib [1 µM] (STI)	13.7	0.8	ns		65/30	4
	GRU102 (nAD)	-	10.0	0.6		<0.0001	52/0	6
		Imatinib [1 µM] (STI)	12.6	0.7	0.04	ns	84/30	6
Fig. 3H	NV48 (wt)	-	14.4	0.6			88/41	6
		*abl-1* RNAi	13.4	0.7	ns		78/29	6
	NV49 (nAD)	-	9.6	0.5		<0.0001	76/0	6
		*abl-1* RNAi	12.5	0.7	<0.0001	ns	88/2	6
Fig. 3I	NV48 (wt)	-	14.6	1.0	ns		39/16	3
	NV50 (*abl-1* KO)	-	12.9	1.2		ns	31/11	3
	NV49 (nAD)	-	9.0	0.8	0.02	0.001	30/0	3
	NV51 (nAD;*abl-1* KO)	-	11.7	1.0		0.01	33/5	3
Fig. S2C	NV48 (wt)	-	14.6	1.0			39/16	3
		Imatinib [1 µM] (STI)	15.0	0.8	ns		51/25	4
	NV49 (nAD)	-	9.0	0.8		<0.0001	30/0	3
		Imatinib [1 µM] (STI)	11.2	0.8	0.007	0.002	59/12	4
	NV51 (nAD;*abl-1* KO)	-	11.7	1.0		0.01	33/5	3
		Imatinib [1 µM] (STI)	12.4	0.7	ns	0.009	64/16	4

^a^Pairwise comparisons using Log-Rank test.

To further investigate the protective effect of Abl suppression against AD pathology, we then looked at another neuronal readout affected by Aβ toxicity. Namely, expression of human Aβ under a muscle specific promoter was previously shown to impair animals’ sensing and habituation (learning) ability [35]. Consistent with previous findings we observed that wild-type animals have normal chemosensory function (AWA neurons-mediated attraction towards benzaldehyde) suppressed after training with pre-exposure to the same compound (Fig. 3J–L, first two black bars). Instead, we found that similar to muscle Aβ expression also neuronal Aβ expression significantly impaired animals’ chemotaxis index and completely abolished their learning ability (Fig. 3J–L, compare wild-type and AD black bars). Pharmacological suppression of Abl activity with STI did not affect animals’ basal chemotaxis or learning activity in the wild-type strain while its genetic depletion had a mild but significant impact (Fig. 3J–L, compare pink and black plain bars). Strikingly, Abl suppression restored animals’ chemosensory function as well as learning ability in the AD strain, especially upon genetic depletion (Fig. 3J–L, compare black and pink striped bars). To verify whether STI is actually protecting against Aβ-induced pathology via ABL-1 inhibition, we then couple pharmacological and genetic suppression of Abl in the AD model. Remarkably, in support of a specific protection against Aβ-induced pathology via ABL-1 inhibition, STI did not provide additional beneficial effects in the absence of *abl-1*, in most of the assessed parameters affected by neuronal Aβ expression (Fig. S3A–D; Tables 1, 4).

**Fig. 4:**
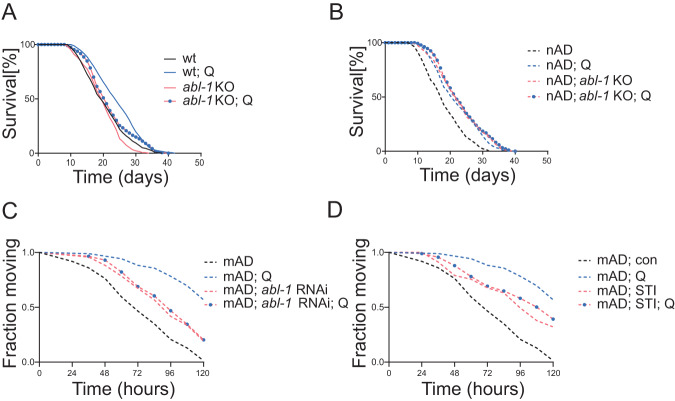
Abl suppression mediates the beneficial effects of quercetin against Aβ-induced pathology. Kaplan-Meier survival curves of wild-type (wt) and *abl-1* knockout (*abl-1* KO) worms (**A**), or neuronal Aβ-expressing (nAD) worms, and nAD crossed with *abl-1* KO worms (**B**), left untreated or treated with Quercetin [100 µM] (Q), see Table 2 for statistics. Fraction of moving worms in response to temperature upshift of muscle Aβ-expressing (mAD) worms, fed OP50(xu363) bacteria transformed with either empty-vector or vector-expressing dsRNA against *abl-1* (*abl-1* RNAi) (**C**), left untreated or treated with Imatinib [1 µM] (STI) (**D**), and left untreated or treated with Quercetin [100 µM] (**C**, **D**), see Table 5 for statistics.

These data clearly support a protective role of Abl suppression against human Aβ toxicity. Moreover, they indicate that Abl depletion provides specific beneficial effects in the Aβ compromised background whilst not affecting neuromuscular parameters (e.g., sensory or locomotion abilities) in otherwise wild-type animals, which differs from the more generic beneficial effects promoted by quercetin (Fig. 1). In further support of a specific protective effect, *abl-1* knock-out per se did not affect lifespan in the otherwise *C. elegans* wild-type strain (Fig. 4A; Table 2) while extending lifespan in the nAD strain (Fig. 4B; Table 2). Most importantly, while quercetin extended lifespan in both the wild-type and the nAD strain, it could not do so in the in the absence of *abl-1* in none of the two strains (Fig. 4A, B; Table 2).

To further support the protective effect of quercetin against Aβ expression via Abl suppression, and exclude non-specific effects ascribed to strain background, we then moved to the *C. elegans* strain with muscle Aβ overexpression. Remarkably, we found that either quercetin alone or genetic or pharmacological *abl-1* suppression, significantly rescued also the paralysis induced by muscle Aβ overexpression (Fig. 4C, D; Table 5). Moreover, consistent with the lifespan results in the nAD strain, the protective effect of quercetin against muscle Aβ-induced paralysis was lost in animals with genetic or pharmacological inhibition of ABL-1 (Fig. 4C, D; Table 5).

**Table 5:** Paralysis assay summary.

	Strain	Treatment	Mean paralysing time (h)	Standard Error	*P* vs untreated^a^	*P* vs (Q)	Total/Censor	*N*
Fig. 4C-D	GMC101 (mAD)	-	75.4	3.2			86/8	3
		Quercetin [100 µM] (Q)	109.4	2.1	<0.0001		92/60	3
		*abl-1* RNAi	93.9	2.8	<0.0001		86/25	3
		*abl-1* RNAi; Quercetin [100 µM] (Q)	91.4	2.8	0.000	<0.0001	95/27	3
		Imatinib [1 µM] STI	91.7	3.1	<0.0001		93/34	3
		Imatinib [1 µM] STI; Quercetin [100 µM] (Q)	96.3	3.0	<0.0001	0.004	91/39	3

^a^Pairwise comparisons using Log-Rank test.

Overall, in strong support of the beneficial role of Abl suppression, data described so far clearly showed that genetic or pharmacological approaches respectively reducing Abl expression or activity, rescue different animals’ defects induced by neuronal or muscular overexpression of toxic human Aβ in *C. elegans*. Moreover, they revealed that quercetin provides protection against Aβ-induced toxicity via ABL-1 suppression.

### Quercetin reduces Aβ secretion in mammalian cells

**Fig. 5:**
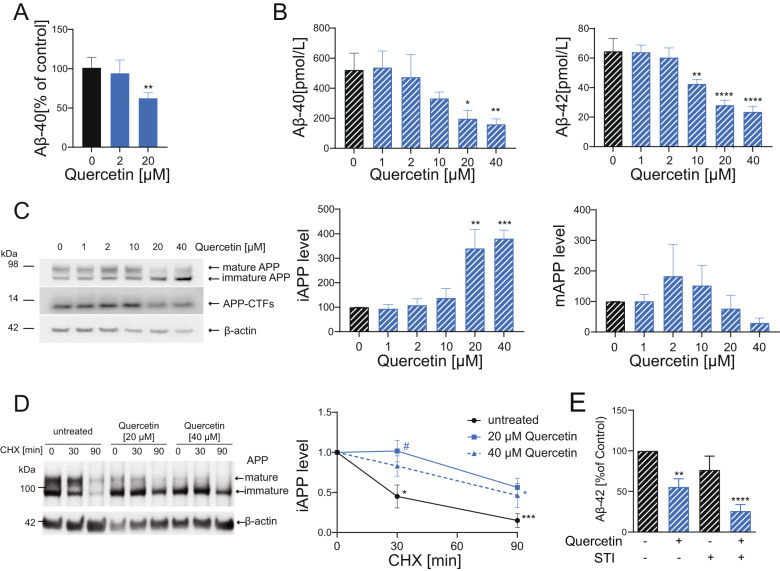
Quercetin reduces Aβ secretion in mammalian cells. **A** Mouse cortical neurons were treated at 3 days in vitro with Quercetin [0–20 µM] for 24 h. Level of Aβ40 was determined by ELISA. Values were normalized to the Aβ level detected in conditioned medium of control cells (*N* = 4). **B** HEK293 expressing hAPP695^wt^ cells were incubated with increasing concentrations of Quercetin [0-40 µM]. 24 h post-exposure secreted Aβ40 and Aβ42 load was evaluated by ELISA (*N* = 3). **C** The level of mature APP (mAPP), immature APP (iAPP) and APP-CTFs were assessed in cellular membrane fraction by Western Blot analysis. Data were normalized to the level of β-actin and respective control (*N* = 4). **D** HEK293-hAPP695^wt^ cells were treated with Quercetin [20 and 40 μM] for 24 h and subsequently exposed to CHX [40 μg/mL] for 0, 30, or 90 min. The level of mature APP (mAPP), immature APP (iAPP) were assessed in the cell lysates by Western Blot analysis. Data were normalized to the level of β-actin and respective control (*N* = 3). **E** HEK293T stably transfected with hAPP695^wt^ were treated for 24 h with Quercetin [20 µM] or Imatinib [10 µM]. Level of Aβ42 was measured by ELISA. Values were normalized to control (*N* = 3). Statistical analyses performed by paired Student’s *t* test *P < 0.05; **P < 0.001; ***P < 0.001 versus untreated condition; while 2-way ANOVA (Tukey´s multiple comparisons test) was used in (**D**), *P < 0.05; ***P < 0.001 versus time 0; #*P* < 0.05 versus untreated at the same time point.

We then sought to address the potential beneficial effects of quercetin also in mammalian cellular models. Aβ toxic peptide derives from pro-amyloidogenic proteolytic processing cleavage of the APP. The amount of secreted Aβ peptide is therefore a very sensitive readout correlating with disease pathology and it represents an important endpoint to evaluate the potential protective or detrimental properties of interventions for AD pathogenesis. Consistent with a protective effect, quercetin treatment reduced Aβ secretion from primary murine cortical neurons (Fig. 5A). We then took advantage of cells stably expressing a wild-type variant of APP (APP695) and found that quercetin reduces both Aβ_1-40_ and Aβ_1-42_ secretion in a dose-dependent manner (Fig. 5B). Reduced amount of Aβ secretion may be primarily ascribed to reduced APP levels or pro-amyloidogenic cleavage or increased Aβ degradation. We thus first analyzed the expression levels of iAPP and mAPP as well as its cleaved products CTFs. Western blot analysis indicated that quercetin significantly increases the level of iAPP in a dose-dependent manner, with a consequent but not significant decrease in its mature form as well as no changes in the CTFs amount (Fig. 5C). Moreover, cycloheximide pulse-chase experiments revealed no altered degradation pattern of mAPP while showing increased stability of iAPP (Fig. 5D), indicating quercetin may impact on processes which regulate iAPP maturation and/or degradation.

Since phosphorylation by Abl was shown to influence both APP maturation and Aβ-degradation pathways [31, 32, 36] we then wondered whether Abl depletion may mediate the beneficial effects of quercetin also in mammalian cells. Interestingly, quantification of Aβ secretion from APP overexpressing cells revealed that quercetin significantly reduces the amount of secreted Aβ (Fig. 5E). This effect was strengthened by the co-treatment with STI, which alone had however negligible effects on the amount of secreted Aβ (Fig. 5E). While the lack of STI effect alone on Aβ secretion could be a dose dependent effect, this data might instead imply that Abl suppression in mammalian cells may protect against Aβ toxicity cooperating with quercetin on different Aβ related features (production/degradation/toxicity). Consistent with this possibility while quercetin alone did not affect the amount of CTFs expression, STI alone has been shown to promote APP non-amyloidogenic cleavage and alter CTFs generation [32]. Data collected so far suggest that Abl inhibition and quercetin may differentially or in parallel impact on APP processing and Aβ toxicity in mammals. Nonetheless, considering that *C. elegans* APP related protein (APL-1) does not contain an Aβ sequence and the available *C. elegans* AD models rely on transgenic expression of the human Aβ toxic peptide [3, 14], the common beneficial effects of quercetin in cells and *C. elegans* must involve Abl-regulated pathways impacting on Aβ levels and/or toxicity (rather than on APP processing).

### Autophagy mediates quercetin-induced Abl-suppression and Aβ-protection

**Fig. 6:**
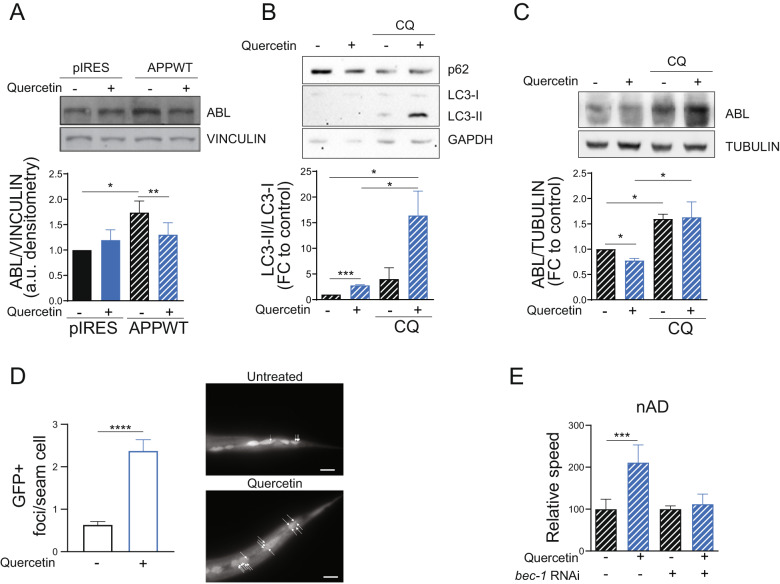
Autophagy mediates quercetin-induced Abl-suppression and Aβ-protection. Immunoblot and densitometric analyses of HEK293T-pIRES and HEK293T-APP^WT^ cells upon Quercetin and Cloroquine treatments. **A** HEK293T-APP^WT^ cells shows a significant upregulation of ABL compared to HEK293T-pIRES cells. Quercetin [20 mM] treatment for 24 h significantly downregulates ABL in HEK293T-APP^WT^ cells. HEK293T-APP^WT^ cells were pre-treated with Cloroquine (CQ) [10 mM] 1 h before Quercetin to assess quercetin-dependent autophagic induction. **B** ABL protein is significantly accumulated in CQ conditions. **C** Vinculin, GAPDH and Tubulin were used as loading control. Statistical analyses performed by paired Student’s *t* test *P < 0.05; **P < 0.001; ***P < 0.001. **D** Number of autophagosomal GFP+ foci in seam cells of L3 larvae in a *C. elegans* strain expressing the GFP under the *lgg-1* promoter fed empty vector expressing HT115(DE3) bacteria, either left untreated or treated with Quercetin [100 µM]. Bar plots represent mean ± SEM (*N* = 3, *n* ≥ 30). Statistical analyses performed by unpaired Student’s *t* test ****P < 0.001. On the right side of the bar plot, representative pictures of the *C. elegans* seam cells used for the measurement are shown. Arrows indicate autophagosomes. Scale bar 20 µm. **E** Relative speed of nAD strain fed, starting from L4, HT115(DE3) bacteria transformed with either empty-vector or vector-expressing dsRNA against *bec-1* (*bec-1* RNAi), bar graphs shown mean ± SD of the normalized values to quercetin untreated conditions (*N* = 3, *n* ≥ 99). ***P < 0.001 calculated with 2-way ANOVA (Tukey´s multiple comparisons test).

To further investigate the role of Abl in the protective effect of quercetin, we then assessed its expression in cells stably transfected with an empty vector or with APP^wt^. Coherently with the literature [28, 37] we confirmed that Abl expression is increased by APP overexpression and most notably found that quercetin significantly reduces its expression (Fig. 6A). Interestingly, quercetin did not modulate Abl transcript expression neither in mammalian cells nor in *C. elegans* (Fig. S4A, B). The antiaging effects of other phenolic natural compounds (e.g., resveratrol, dimethoxychalcone) have been often ascribed to induction of autophagy [38, 39], the major cellular recycling pathway. Moreover, impaired autophagic flux has been found in mammalian as well as *C. elegans* AD models and the autophagy-regulatory gene beclin was once reported to be required for quercetin protective effect against muscle Aβ-induced paralysis in *C. elegans* [20, 30, 40]. Thus, we wondered whether activation of autophagy by quercetin could mediate Abl depletion and ultimately its protective effects in vitro (reduced Aβ secretion) and in vivo in *C. elegans* (reduced Aβ toxicity). Consistent with this possibility, using the autophagy inhibitor chloroquine (CQ) we clearly showed that quercetin induces autophagy in cells stably expressing APP (Fig. 6B). Most notably, quercetin could not reduce Abl expression upon blockage of autophagy with CQ (Fig. 6C).

Consistent with cell data, we could show that quercetin induces autophagy in the nematode *C. elegans*, revealed as an increased number of LC3/LGG-1 foci in the seam cells of animals L3 larvae (Fig. 6D). Most importantly, in further support of quercetin protective effects being mediated by autophagy, silencing of beclin (*bec-1*), a central autophagy regulatory gene, in *C. elegans*, completely prevented quercetin beneficial effects on motility in animals overexpressing toxic Aβ in the neurons (Fig. 6E). Somewhat unexpectedly, not only *bec-1* RNAi prevented the protective effects of quercetin against Aβ-induced toxicity, but it also significantly ameliorated animals’ motility in the absence of quercetin (Fig. S4C). Given the autophagy blockage observed in the different AD models [30, 40], our results imply that preventing the formation of the autophagosomes (via beclin depletion) either prompts the induction of compensatory systems facilitating Aβ degradation, or prevents the accumulation of insoluble toxic aggregates or of proteins that would favor Aβ toxicity. Our results in mammalian cells (Fig. 6B, C) suggest that Abl accumulation could be at least in part responsible for the detrimental effect of autophagy blockage in the different AD models.

Furthermore, similar to what we observed in the muscle Aβ overexpressing strain (Fig. 4C, D), *abl-1* knock-out suppressed the motility defect in the nAD strain thus masking the beneficial effect of quercetin (Fig. S4D). Remarkably, *abl-1* depletion also completely prevented the beneficial effects of *bec-1* silencing against Aβ-induced toxicity (Fig. S4D). This implies that autophagy activation on the one hand mediates quercetin-induced depletion of ABL-1 and, on the other hand, it participates in the protective effect elicited by ABL-1 suppression, as also recently shown by pharmacologic inhibition of Abl in AD mice [30]. In support of autophagy being a commonly regulated pathway between quercetin treatment and ABL-1 depletion, a closer analysis of the 171 genes commonly affected between the two interventions in *C. elegans*, revealed an enrichment of genes targeted by TFEB/hlh-30 (Fig. S4E), a master regulator of the autophagy process [41].

Overall, our in vitro cellular data coupled with the in vivo *C. elegans* behavioral assays, reveal that quercetin protection against Aβ toxicity is mediated by reduced Abl expression through induction of autophagy. Moreover, they support the notion that autophagy activation concurrently specifies the beneficial effects of Abl suppression. Quercetin would therefore overcome Aβ-induced blockage of autophagy counteracting the vicious cycle which favors Abl accumulation.

## Discussion

Plant-derived compounds, such as polyphenols and carotenoids found in food and beverage, are emerging as promising interventions to promote healthy aging and delay the development and progression of different age-associated disorders [18, 19, 42]. Their anti-aging properties have been largely discovered in short-lived model organisms such as the nematode *C. elegans* but the underlying molecular mechanism, besides their antioxidant activity is poorly understood [24, 43]. In this work, we initially tested the potential protective effects of four different natural compounds against *C. elegans* aging and AD, the most prevalent age-associated disease. While EGCG, lutein and lycopene primarily protected animals from overexpressing human toxic Aβ in the neurons, quercetin promoted more general or systemic beneficial effects irrespective of neuronal damage. In support of our findings, another study showed that lycopene protects *C. elegans* against muscle-Aβ induced paralysis and reduces ROS levels and apoptosis in APPsw (a mutant form of APP which favors its amyloidogenic processing) cells only upon peroxide or copper treatment (but not in basal conditions) [44]. Some studies also indicated lutein and EGCG preferentially display beneficial effects in compromised *C. elegans* backgrounds [45, 46], while others have shown the compounds increase stress resistance and extend lifespan also in wild-type animals [47, 48]. These seemingly conflicting results can be explained by different exposure scenarios: bimodal dose-dependent effects have been often reported with dietary interventions [47, 49]; different bacterial types used as food source (e.g., HT115 vs OP50; dead vs alive bacteria) can influence animals phenotypes and behaviors in basal conditions, as well as compounds metabolism and therefore their biological effects on lifespan and age-associated features [50–52]; vehicles, methods and age of administration may add further variability [53–55]. For instance, we here observed that different experimental paradigms (e.g., ±solvent) slightly affect resistance to stress and attraction to food in the nAD strain compared to wild-type animals. Moreover, differently from the original study where OP50 bacteria were used as food source [23], here we used HT115 bacteria plus vehicle, which did not shorten the lifespan of the nAD compared to wild-type animals.

The protective effect of quercetin against different type of stressors and aging has been instead consistently recognized in different model organisms [24–26] but its protection against age-associated neuropathologies has not been actively investigated. Of note, quercetin is one of the most extensively investigated polyphenols for its antiaging, anticancer and anti-inflammatory properties, and clinical trials have evaluated its effect against chronic diseases [18, 42, 56, 57]. In this study, we further expanded its beneficial effects against neuropathologies and showed that quercetin reduces Aβ secretion in mammalian cells and promotes health and lifespan in wt *C. elegans* as well as in animals overexpressing human toxic Aβ either in neuronal or in muscle cells. Our coupled in vivo and in vitro results indicate that quercetin’s beneficial effect against Aβ toxicity is mediated by autophagy activation. Autophagy activation has been already shown to protect against toxicity induced by Aβ, polyglutamine aggregates or overexpression of a mutant superoxide dismutase in the respective *C. elegans* disease models, namely AD [40], Huntington disease (HD) [58] and Amyotrophic Lateral Sclerosis (ALS) [59]. Interestingly, along with other degradation pathways, autophagy is involved in shaping up synaptic structure and function contributing to memory formation [60, 61]. Most notably, synaptic alterations have been described in a few *C. elegans* studies during aging [62, 63] (including the nAD strain used in this work—*our unpublished observation*) and impairments of synaptic plasticity is an initial event underlying the memory loss and cognitive decline typically found in AD patients [64–67]. Thus, hampered autophagy during aging or in the context of AD is expected to lead to synaptic dysfunction-induced memory loss and quercetin could help counteracting this early neuronal defect.

While the beneficial effects of quercetin, or of other natural compounds, against aging or stress have been already ascribed to autophagy induction across species [19, 38, 68], in this study we identified for the first time a specific autophagy target, Abl tyrosine kinase, necessary for its protective effect in the context of AD. Our RNA-Seq analysis indicates genes affected by quercetin are enriched in protein kinases and phosphatases and quercetin was shown to inhibit a panel of different cancer-relevant kinases [69]. Several kinases are implicated in aging and age-associated pathologies across species (e.g., the insulin-like receptors, the target of rapamycin) [1], and protein kinase inhibitors were predicted among the highest ranked drugs in search of anti-aging compounds [70]. Remarkably, different kinases have been also shown to orchestrate neuronal functions and synaptic plasticity with Abl being implicated in the regulation of neuronal biogenesis, synaptic formation and functionality [71, 72]. Consistent with its role in the nervous system, aberrant Abl expression or activity have been found in mammalian models of different neurodegenerative disease including AD [37, 73, 74]. Most notably, recent studies in cellular and mice models pointed to Abl tyrosine kinase as a promising therapeutic target for AD [30–32]. Accordingly, we found that Abl suppression via genetic (RNAi or knock-out) or pharmacological (Imatinib) treatments, also has beneficial effects in *C. elegans* AD models and provided evidence that Imatinib works through Abl also in *C. elegans*, as its effect is masked in the *abl-1* knock-out strain. Furthermore, we found that reduced Abl expression via autophagy is necessary to mediate the protective effects of quercetin. The specific mechanisms through which quercetin-induced autophagy leads to Abl degradation remains to be identified. Nonetheless, it is interesting to note that, different from quercetin, Abl suppression alone neither impacted on Aβ secretion in mammalian cells nor improved lifespan or neuromuscular parameters in wt *C. elegans* (compared to its effect in the AD strains). While these results may be ascribed to specific experimental conditions (e.g., cells-specific effects, compounds dosage or vehicles), they may as well imply that the two interventions, on top of their convergent activity, also cooperate to protect against Alzheimer pathology through independent mechanisms. This notion is consistent with their different effect we observed on APP processing in mammalian cells. Moreover, it is supported by the fact that *C. elegans* APP related protein (APL-1) does not contain an Aβ sequence and available AD models rely on transgenic expression of the human Aβ toxic peptide [3, 14], rather than on increased pro-amyloidogenic processing of APP. Activation of different pathways may thus concur to protect against Aβ toxicity in parallel or downstream of Abl-suppression upon quercetin treatment.

Abl inhibition has been shown to impact on APP processing in mammals in different ways and especially through modulation of APP intracellular domain (AICD) phosphorylation, expression or activity, thus ultimately impacting on Aβ levels and toxicity [36, 75–78]. Also, some natural compounds, including quercetin, were sporadically reported to reduce APP pro-amyloidogenic processing [79–81]. While modulation of APP processing cannot explain the beneficial effect of quercetin or Abl inhibition in *C. elegans* AD models, some of the enzymes involved in APP processing in mammals are conserved in nematode (e.g., Fe65/*feh-1*, neprilysin/*nep-1* [3, 82, 83]) and, if affected by the two interventions, may still impact on Aβ degradation or toxicity. Moreover, natural compounds as well tyrosine kinases inhibitors could activate cytoprotective responses (e.g., mtUPR, mitophagy, Nrf2), which in turn counteract Aβ toxicity [23, 47, 84–90]. Remarkably, in this work, we found that autophagy is required on the one hand for quercetin to reduce Abl expression, and on the other hand to mediate the protective effect of Abl suppression in *C. elegans*. In support of our study others have described a beneficial effect of Abl inhibition in mammalian models of neurodegenerative disorders where Abl is aberrantly expressed/activated and the lysosomal/autophagic function is concurrently compromised [30, 91–93]. The exact mechanism through which Abl is impacting on autophagy remain to be fully elucidated but our analysis of the 171 genes in common between quercetin-treated and Abl-depleted animals pointed to genes controlled by TFEB/*hlh-30*, a master regulator of autophagy [41]. In support of our findings, a handful of studies also described enhanced TFEB transcriptional activity by Abl inhibition in other neurometabolic disorders such as ALS or Niemann-Pick type C disease [94, 95]. Follow up investigations on the gene expression uniquely or commonly modulated by quercetin and Abl suppression will shed light on specific targets required for their protection against Aβ toxicity.

In conclusion, we propose that quercetin, by stimulating autophagy, would reset an appropriate level of Abl tyrosine kinase whose abnormal expression contribute in a vicious cycle to the autophagy blockage observed in different AD models. How autophagy reduces Abl expression and how in turn autophagy activation by Abl (possibly in a TFEB-dependent manner) protects against Aβ toxicity remain to be fully elucidated. Nonetheless, the exploitation of natural compounds rather than drugs reducing Abl activity, has clear advantages, avoiding the appearance of side effects while promoting broader beneficial effects. Interestingly, the combination of quercetin plus dasatinib (a non-specific pharmacological inhibitor of Abl and Src tyrosine kinases) has been proposed as treatment to promote healthy aging thanks to their senolytic effect i.e., the ability of the treatments to specifically kill senescent cells [96–99]. While there is no evidence that senescent cells killing plays a role in *C. elegans* aging, our findings suggest new mechanisms may underly the beneficial effect of combining quercetin and kinases inhibitors, which most likely go beyond their classical senolytic activity. Overall, we provide strong support for exploiting *C. elegans* as an excellent in vivo model organism to identify possible AD pathogenic targets and therapeutics and disantangle their underlying mode of action.

## Material and methods

### Statistical analysis

The data analysis was done using Prism V9 software (GraphPad Software Inc., San Diego, USA) and R programming language (http://www.R-project.org). The statistical tests used for each experiment are detailed in the figures’ legends. *N* = number of independent biological replicas; *n* = sample size. The sample size for each experiment was selected according to existing literature data on comparable published experiments.

### *C. elegans*

#### *C. elegans* strains and culture conditions

The following strains were used in this study:

GRU101: *gnaIs1* [myo-2p::YFP],

GRU102: *gnaIs2* [myo-2p::YFP + unc-119p::Abeta1-42],

XR1: *abl-1(ok171)*,

NV48: wild type isolated from crossing XR1 with GRU102

NV49: *gnaIs2* [myo-2p::YFP + unc-119p::Abeta1-42] isolated from crossing XR1 with GRU102

NV50: *abl-1(ok171)*, isolated from crossing XR1 with GRU102,

NV51: *gnaIs2* [myo-2p::YFP + unc-119p::Abeta1-42]; *abl-1(ok171)*,

GMC101: *dvIs100* [unc-54p::Abeta-1-42::unc-54 3′-UTR; mtl-2p::GFP]

CL995:*adIs2122*(Plgg-1::GFP::lgg-1 + pRF4)

JKM2: *Is* [rgef-1p::Signalpeptide-Abeta(1 − 42)::hsp-3(IRES)::wrmScarlet-Abeta(1−42)::unc-54(3′UTR) + rps-0p::HygroR]

JKM3: *Is* [rgef-1p::hsp-3(IRES)::wrmScarlet::unc-54(3′UTR) + rps-0p::HygroR]

NV57: *Is* [rgef-1p::Signalpeptide-Abeta(1 − 42)::hsp-3(IRES)::wrmScarlet-Abeta(1−42)::unc-54(3′UTR) + rps-0p::HygroR]; *abl-1(ok171)*

NV58: *Is* [rgef-1p::hsp-3(IRES)::wrmScarlet::unc-54(3′UTR) + rps-0p::HygroR]; *abl-1 (ok171)*

NV strains were specifically generated for this work.

All strains were maintained and kept synchronized by egg lay at 20 °C on Nematode Growth Media (NGM) agar supplemented with Escherichia coli OP50 unless otherwise indicated.

#### RNA-mediated interference (RNAi)

Genes of interest were silenced by feeding *E. coli* HT115(DE3) or OP50(xu363) expressing plasmids transformed for the specified gene, empty vector was used as control. Worms were treated with RNAi expressing bacteria from eggs till the end of the experiment, unless otherwise indicated.

#### Chemical treatments

Quercetin (Q4951 Sigma-Aldrich), Lutein (PHR1699 Sigma-Aldrich), Lycopene (PHR1770 Sigma-Aldrich), Epigallocatechin gallate (ECGC) (E009 TransMit) where dissolved in a solution of Dimethylsulfoxid (DMSO, 276855 Sigma-Aldrich) containing 1% of Tween 80 (P1754 Sigma-Aldrich) and mixed with bacteria to the following concentrations: Quercetin 100 µm, lutein µM100, lycopene 4.6 µM, ECGC 0.64 µM. Control worms were fed bacteria containing the same amount of solvent (0.5% DMSO plus 0.005% Tween 80) used to prepare the above compound. Serotonin (H9523 Sigma-Aldrich) treatment: worms were incubated in 200 µl of 10 mM Serotonin diluted in S-Basal. Imatinib (STI-57, SML1027 Sigma-Aldrich), was dissolved in H_2_O and mixed with the bacteria to a final concentration of 1 µM.

#### Body bends

The movement of adult worms (3 days after egg-lay) was scored on 5 µl of S-Basal. Single worms were transferred into the 5 µl S-basal drop and left adapt for 10–15 s before counting the bends for 20 s. At least ten worms were counted for each replicate.

#### Lifespan

Age synchronized populations of 60–80 worms were used to start the lifespan analysis. To avoid cross-generation contamination, animals were transferred on fresh plates every day during the fertile phase afterward every other day. Animals not able to move upon pick-prodding and with no pharyngeal pumping were scored as dead Animals were scored as not moving when no sinusoid locomotory activity was observed anymore upon prodding. Survival analysis was performed in OASIS 2 [100] using the Kaplan Meier estimator. Statistical differences were evaluated using the log-rank test between the pooled population or worms and *p* values were adjusted for multiple comparisons by Bonferroni method.

#### Heat shock response

The stress resistance of the different strains/treatments was tested by heat shock at 35 °C for 10–11 h. Around 10–15 age synchronized worms were transferred to fresh plates and incubated on 35 °C and the survival was scored manually every single hour until the whole population died. Animals not able to move upon pick-prodding and with no pharyngeal pumping were scored as dead Animals. Survival analysis was performed in OASIS 2 [100] using the Kaplan Meier estimator. Statistical differences were evaluated using the log-rank test between the pooled population or worms and *p* values were adjusted for multiple comparisons by Bonferroni method.

#### Food assay

7 days old worms were used to perform the assay. Briefly, the day before the experiment 6 cm petri dishes containing a modified version of NGM (2% agar, 1 mM CaaCl_2_, 1 mM Mg SO_4_), were marked with 2 dots about 4.5 cm apart, in one of the points 50 µl of freshly grown op50 were seeded. Prior to proceeding with the assay worms were washed 3–4 times with S-Basal and then placed on the opposite dot from the bacteria. The percentage of animals on food was calculated after 2 h from placing the worms on the plate. Around 100–150 worms were used in each replicate in three independent experiments

#### Chemotaxis assay

3 days old worms were used for the assay on 9 cm petri dish containing: 2% agar, 1 mM CaCl_2_, 1 mM Mg SO_4_. Before proceeding with the assay, worms were starved for 2 h either with (trained) or without Benzaldehyde [1%]. Meanwhile, 1 µl of 1% Benzaldehyde and 1 µl of 95% Ethanol, were spotted on the test plates along the diameter, spaced 3.5 cm from the center.

After the starvation period, 40–50 worms were placed on the geometric center of the test plates. The assay plates were incubated for 1 h and a chemotaxis index (CI) was scored manually using the following formula (CI = ([(#benzaldehyde)-(#Ethanol)]/[(#Total-#Center)]. #= number of worms of the specified spot. Each experiment was repeated three times [101].

#### Serotonin sensitivity assay

Worms were incubated in a solution of 10 mM Serotonin (hypochlorite) diluted in S-Basal. Briefly, 1-day-old synchronized worms were collected, washed with S-Basal Buffer, and transferred to 96-well plates containing 200 µl of the Serotonin solution. The immobilized worms were then visually scored every 5 min for 20 min [33].

#### Paralysis assay

Nematodes’ eggs were left to develop at 20 °C for 72 h then upshifted at 25 °C to promote the muscle expressed Abeta-1–42 aggregation. After 24 h from the upshift at 25 °C paralyzed worms were scored manually every 12 h. Nematodes were considered paralyzed if they were unable to move their entire body, either on their own or when prompted by touch. For each assay, around 30 worms for conditions were used in three independent replicates. Paralysis analysis was performed in OASIS 2 [100] using the Kaplan Meier estimator. Statistical differences were evaluated using the log-rank test between the pooled population of worms and *p* values were adjusted for multiple comparisons by Bonferroni method.

#### Relative speed measurement

To measure the relative speed, 25 s movies of worms crawling on plates were recorded and analyzed with Fiji plugin wrMTrck [102, 103]. 3 days old worms, were recorded using a Raspberry HQ camera V1.0 2018, connected to a Zeiss Stereo Discovery.V8 stereomicroscope. All videos were captured using the same hardware and software settings.

The raw videos were converted to 8-bit and segmented using Fiji, then analyzed using wrMTrck. For each condition, two plates containing ~20–25 worms were used to record the movies, and each experiment was repeated three times.

#### Autophagy quantification

The number of autophagic foci was quantified in L3 larvae expressing *Plgg-1*::GFP::LGG-1 worms placed on 2% agarose pads using a Zeiss Axioplan II microscope at a magnification of 630-fold. Three separate biological experiments were conducted and the average number of foci per seam cell (±standard error of the mean) was calculated from a minimum of eight animals per condition.

#### Aβ aggregation quantification in nematodes

Day 4 and day 7 old nematodes were used to quantify Aβ aggregation by fluorescence lifetime imaging (FLIM) in three biological replicates with at least ten nematodes each [34, 104]. Nematodes were anesthetized with 250 mM NaN_3_, placed onto 3% agar pads and imaged with a Zeiss LSM880 confocal microscope. The measurements were recorded using Time-correlated single-photon counting with 40× oil immersion objective, digital zoom of 1.8-fold. The pulsed excitation laser at 40 MHz was set for 485 nm to measure emission between 575 and 620 nm. The measurements were carried out until ~3000 photons were acquired. Data were recorded using SymphoTime 64 and fitted assuming mono-exponential decays with FLIMFIT 5.1.1. software [105]. Statistical differences between strain and ages were calculated using two-way ANOVA + Tukey post hoc test in GraphPad Prism 9. was calculated using 2-way ANOVA + Tukey post-hoc test in GraphPad Prism 9.

#### RNA-Seq

##### RNA extraction and processing

RNA was extracted from nematodes collected on day 3 from 2 large NGM plates seeded with HT115 transformed with the empty vector L4440. Approximately 1000 worms were collected per condition and were frozen in nuclease-free water at −80 °C for further processing. Lysates were prepared using a tissue homogenizer “precellys 24” (Bertin Technologies) and 1:1 v:v 1 mm glass beads (Biospec Cat. No. 11079110) were added to the worms’ pellet along with lysis buffer. The samples were shaken for 30 s at 6000 rpm and then placed on ice for 1 min, repeated three times. Total RNA was extracted using the RNeasy Plus Mini Kit (Qiagen- 74134) according to the manufacturer’s instructions. Five different biological replicas were used for each condition. NV48 (nAD) and NV51(*abl-1* KO) were either left untreated or treated with Quercetin [100 µM] starting from eggs.

DNase digested total RNA samples used for transcriptome analyses were quantified (Qubit RNA HS Assay, Thermo Fisher Scientific, MA, USA) and quality measured by capillary electrophoresis using the Fragment Analyzer and the “Total RNA Standard Sensitivity Assay” (Agilent Technologies, Inc. Santa Clara, USA). All samples in this study showed high quality RNA Quality Numbers (RQN; mean = 10.0). The library preparation was performed according to the manufacturer’s protocol using the Illumina® “TruSeq Stranded mRNA Library Prep” Kit. Briefly, 350 ng total RNA were used for mRNA capturing, fragmentation, the synthesis of cDNA, adapter ligation and library amplification. Bead purified libraries were normalized and finally sequenced on the HiSeq 3000/4000 system (Illumina Inc. San Diego, CA, USA) with a read setup of SR 1×150 bp. The Illumina bcl2fastq tool (v2.20.0.422) was used to convert the bcl files to fastq files as well for adapter trimming and demultiplexing.

##### RNA-Seq analysis

Data analyses on fastq files were conducted with CLC Genomics Workbench (version 20.0.4 and 21.0.4, QIAGEN, Venlo, NL). The reads of all probes were adapter trimmed (Illumina TruSeq) and quality trimmed (using the default parameters: bases below Q13 were trimmed from the end of the reads, ambiguous nucleotides maximal 2). Mapping was done against the *C. elegans* (WBcel235.99) (March 26, 2020) genome sequence. After grouping of samples according to their respective experimental condition, the statistical differential expression was determined using the CLC Differential Expression for RNA-Seq tool (version 2.5). The Resulting *P* values were corrected for multiple testing by FDR and Bonferroni-correction. A *P* value of ≤0.05 was considered significant. The RNA-Seq data discussed in this publication have been deposited in NCBI’s Gene Expression Omnibus [106] and are accessible through GEO Series accession number GSE235199.

##### Gene ontology (GO) analysis

In order to perform functional enrichment analysis, the lists of differential expressed genes, which resulted from the RNA-Seq analysis, were input into the on-line tool g:Profiler (10.1093/nar/gkz369) with the default setting for *C. elegans*. The top 20 GO terms were plotted for the specified comparison, sorted by significance were plotted. DEG lists were used to find common DEG between the comparison of interest, the result was plotted as Venn diagram (https://bioinformatics.psb.ugent.be/webtools/Venn/),

The enrichment map, visible inside the Venn Diagram was plotted using Cytoscape [107] and the EnrichmentMap plugin [108] following the methods described in ref. [109]. The genes log fold change of the Venn Diagram intersection, was plotted as heat map using the R package ComplexHeatMap [110].

### Mammalian cells

#### Cell culture and stable cell lines generation

Mouse primary cortical cultures (Fig. 5A) were prepared from P0 brains from C57/Bl6J mice as previously described [111] under the study approval by the Landesamt für Natur, Umwelt und Verbraucherschutz (LANUV, Northrhine Westphalia, Germany, reference number Az. 84-02.05.40.14.138 and Az. 81-02.05.50.17.018). 2 × 10^5^ cells were plated in six-well coated with poly-L-lysine (0.1 mg/ml) and containing Neurobasal medium with B27 supplement (Invitrogen).

Human embryonic kidney (HEK) 293 cells stably overexpressing human wild type APP695 (Fig. 5B–D, HEK293APPwt) kindly provided by Prof. Jochen Walter, Uni Bonn [112], were grown in RPMI1640 (Invitrogen) supplemented with 10% heat-inactivated fetal bovine serum (Perbio) and 100 µg/ml of penicillin/streptomycin (Invitrogen).

Commercially available HEK293T cells (Sigma; Figs. 5E and 6A–C) were grown in Dulbecco’s modified Eagle’s medium (DMEM) supplemented with 10% fetal bovine serum, 100 U/ml penicillin, and 100 mg/ml streptomycin (Sigma-Aldrich). Stable overexpression of pIRES-empty and pIRES-APP695^WT^ vectors were performed by using Polyethylenimine reagent (Tebu-bio), according to manufacturer’s instructions. Hygromicing B (Sigma-Aldrich) was use as selection antibiotic at the concentration of 200 mg/ml.

#### Cell treatments

Primary cortical neurons (2 × 10^5^ cells/well) were treated at day 3 in vitro with Quercetin for 24 h.

HEK293T cells were seeded at the proper density and treated the day after for experiments. Quercetin 20 µM treatment was performed for 24 h; Cloroquine 10 µM treatment was performed 1 h before quercetin. All the reagents were purchased from Sigma-Aldrich.

HEK293APP695wt cells were treated 24 h after seeding at 70% confluence with different concentrations of quercetin (1 – 40 µM) or imatinib (5–20 µM; Sigma-Aldrich) dissolved in DMSO. Control cells were treated with medium containing the highest used amount of solvent (0.1% DMSO). 24 h after treatment, conditioned media were aspirated, centrifuged at 1.200 rpm and supernatants were stored in −20 °C for ELISA analysis. Subsequently, the cellular membranes were extracted and used for western blot analysis.

#### Western blot analyses and amyloid-β ELISA

To assess the turnover of APP, cells treated for 24 h with quercetin as described above and exposed to cyclohexamide (Sigma-Aldrich) [40 μg/mL] for 0 min, 30 min and 90 min. Then, cells were lysed in STEN lysis buffer (1× STEN: 50 mM Tris, pH 7.6, 150 mM NaCl, 2 mM EDTA, 0.2% Nonidet P-40; STEN-lysis buffer, 1% Triton X-100, 1% Nonidet P-40, complete protease inhibitors in 1× STEN) on ice for 30 min and clarified by a 30-min centrifugation at 13,200 rpm.

Total cell extracts were obtained by rupturing cells in RIPA buffer (50 mM Tris–HCl, pH 8, 150 mM NaCl, 1% NP-40, 0.5% sodium deoxycholate, 0.1% SDS, 10 mM NaF, 1 mM sodium orthovanadate) and protease inhibitor cocktail (Roche Applied Science) followed by centrifugation at 14,000 × *g* for 20 min at 4 °C. 30 mg protein extracts were then electrophoresed by SDS–PAGE and blotted onto nitrocellulose membrane (Bio-Rad Laboratories). Cellular membrane preparation: All procedures were carried out at 4 °C. Cells were harvested and resuspended in hypotonic buffer (10 mM Tris, pH 7.3, 10 mM MgCl_2_,1 mM EDTA and 1 mM EGTA) for 10 min on ice. Cells were then homogenized by passing ten times through a 21-gauge needle and centrifuged for 10 min at 100 × *g* to pellet nuclei. The resulting supernatant was centrifuged 30 min at 16,000 × *g*. Crude cellular membrane fractions were lysed in STEN lysis buffer (1×STEN: 50 mM Tris, pH 7.6, 150 mM NaCl, 2 mM EDTA, 0.2% Nonidet P-40; STEN-lysis buffer, 1% Triton X-100, 1% Nonidet P-40, complete protease inhibitors in 1× STEN) and clarified by a 30-min centrifugation at 13,200 × *g*. Upon SDS-PAGE electrophoresis, membrane proteins were transferred to nitrocellulose membrane and detected with the corresponding antibodies.

Primary antibodies used are as follows: anti-p62 (MBL, #PM045); anti-LC3 (8E10) (MBL, #M186-3); anti-ABL (Ab-3) (Sigma-Aldrich, #OP20); anti-Vinculin (13901T) (Cell Signaling Technology, #13901); anti-GAPDH (D16H11) (Cell Signaling Technology, #5174); anti-Tubulin (Sigma-Aldrich, #T5168). The specific protein complex, formed upon incubation with specific secondary antibodies (Bio-Rad Laboratories), was identified using a iBright Imaging Systems (Thermo Fisher Scientific), after incubation with the ECL detection system (Bio-Rad Laboratories). Images were adjusted for brightness and contrast by Fiji analysis software.

APP full-length and APP C-terminal fragments were detected with APP CT antibody (Sigma). The Aβ40 and Aβ42 ELISA were performed according to the manufacturer’s manual (Wako chemicals, Germany).

Uncropped original blots are shown in the Supplemental Material file.

#### Real-time PCR

RNA was extracted by using TRI Reagent (Sigma-Aldrich), in accordance with manufacturer protocol. cDNA was generated starting from 1 mg of total RNA using the SensiFAST cDNA Synthesis KIT (Bioline). Specific primer pairs were designed to amplify unique regions of genes of interest, primers sequences are listed below. RT–qPCR was performed using the SensiFAST SYBR Green Master Mix (Bioline) on a LightCycler 480 System (Roche). Data were analyzed following the 2^−^^ΔΔCt^ method. The fold changes in mRNA levels were determined relative to the control after normalizing to the internal standard actin.

**Table:** 

Genes	Forward	Reverse
*ABL1*	CCAGGTGTATGAGCTGCTAGAG	GTCAGAGGGATTCCACTGCCAA
b-actin	GGGACCTGACTGACTACCTC	ATCTTCATTGTGCTGGGTG

### Supplementary information

Figure S1

Figure S2

Figure S3

Figure S4

Supplementary Figure Legends

Uncropped western blots
