# Supplementary figures and images for: Abl depletion via autophagy mediates the beneficial effects of quercetin against Alzheimer pathology across species
Source: Cell Death Discov. 2023 Oct 14;9:376. doi: 10.1038/s41420-023-01592-x (PMC10576830; doi:10.1038/s41420-023-01592-x)

Figure S1

A

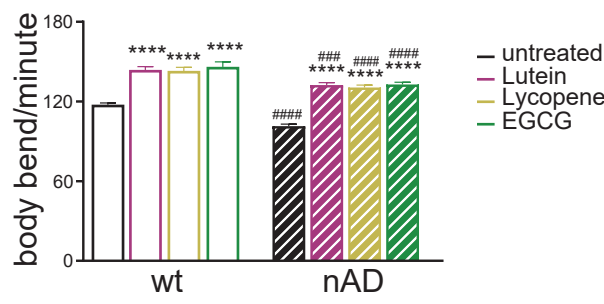

B

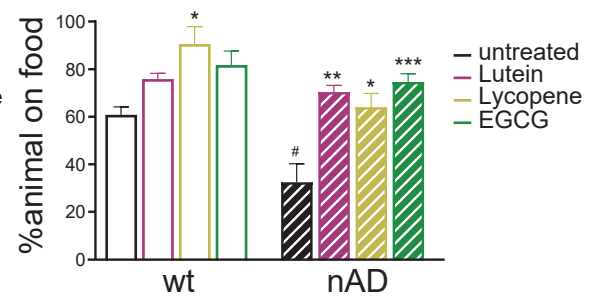

C

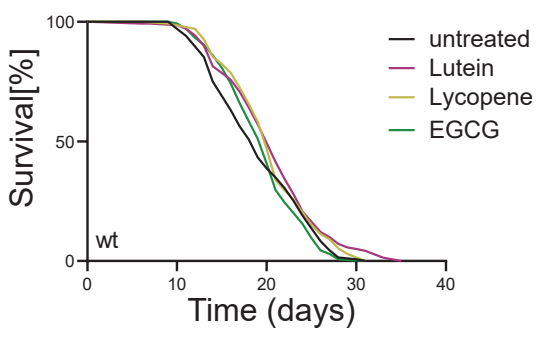

D

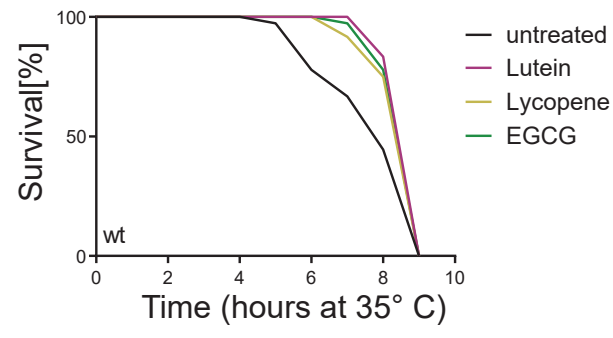

E

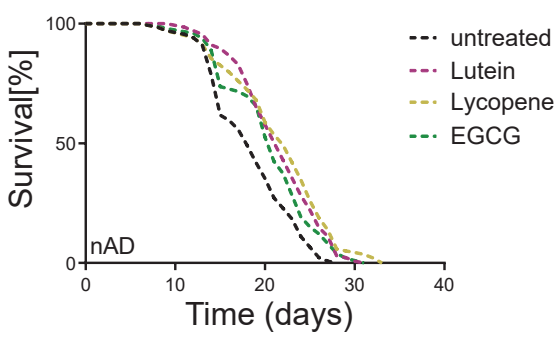

F

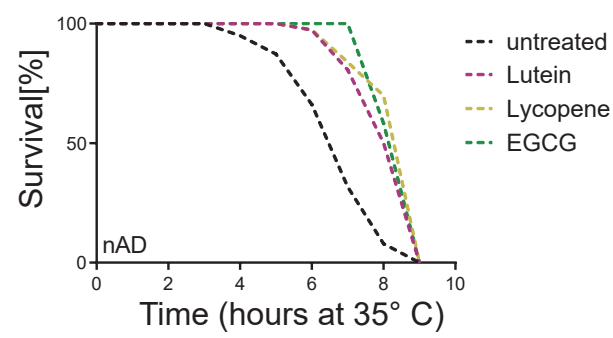

G

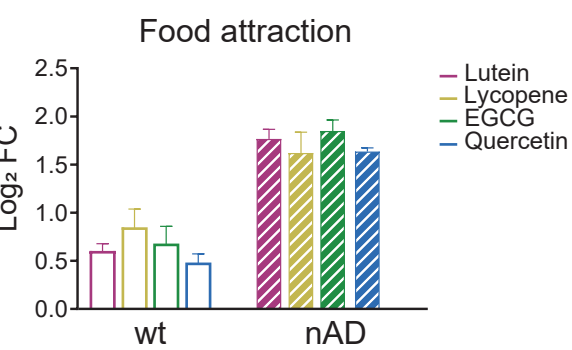

H

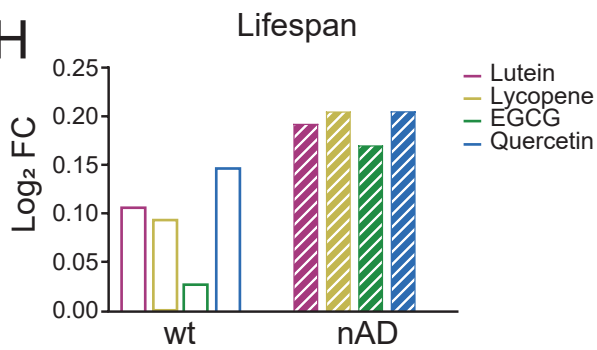

Supplement: Supplementary file 1 — Figure S1 [file 41420_2023_1592_MOESM1_ESM.pdf]

Figure S3

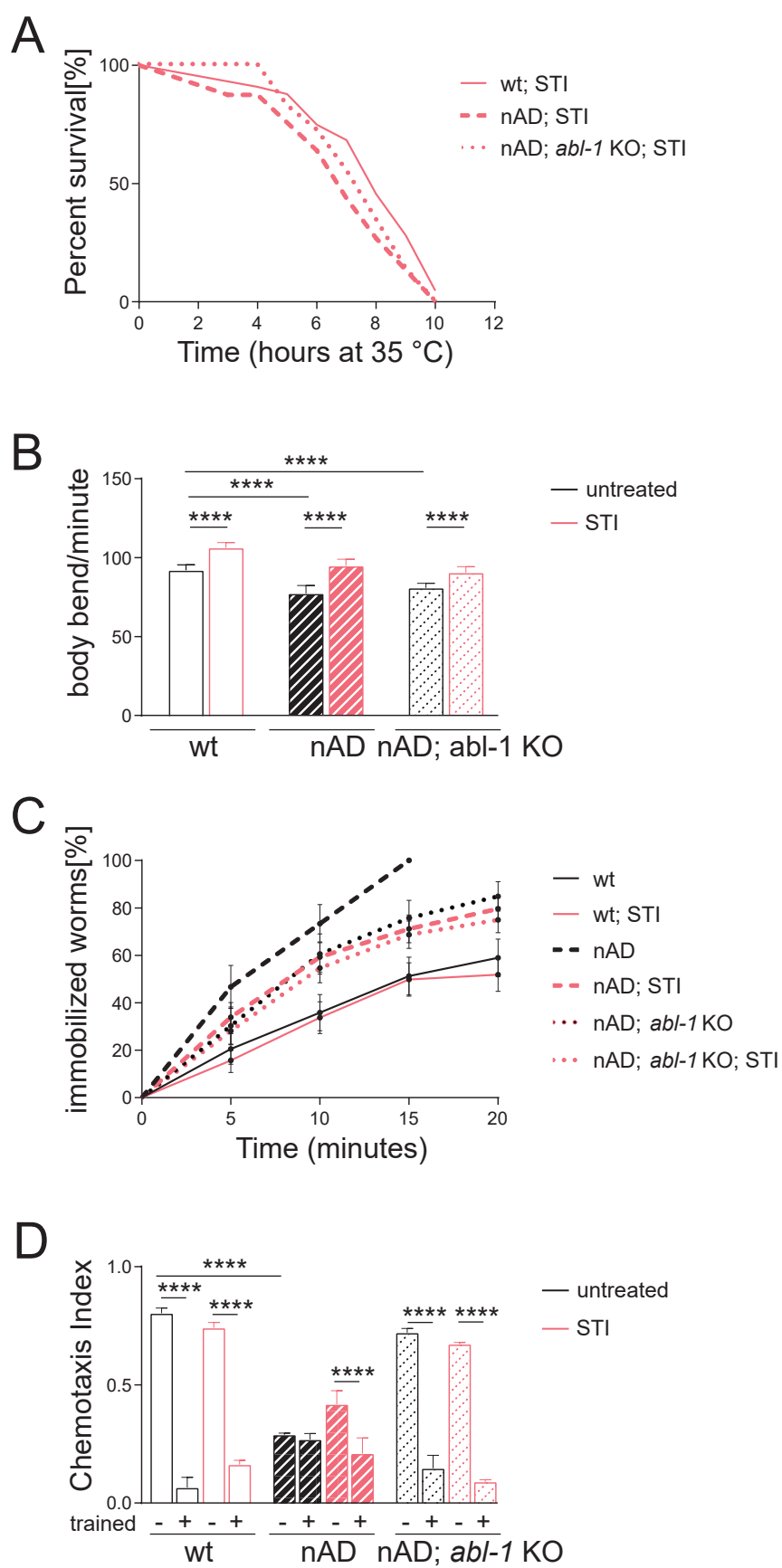

Supplement: Supplementary file 3 — Figure S3 [file 41420_2023_1592_MOESM3_ESM.pdf]

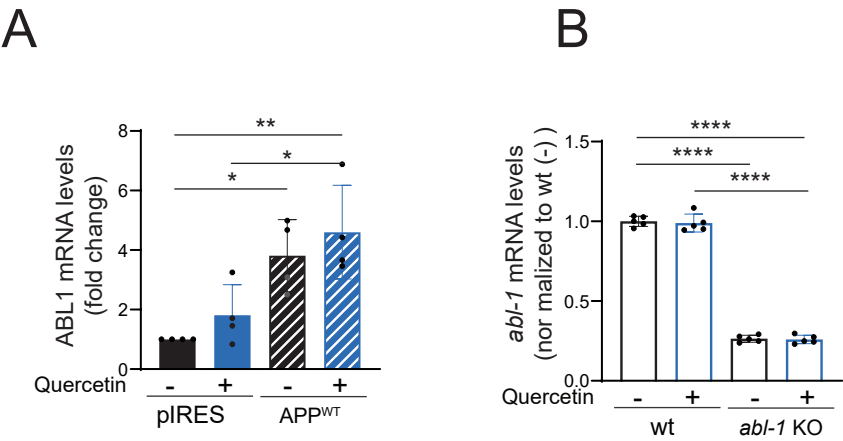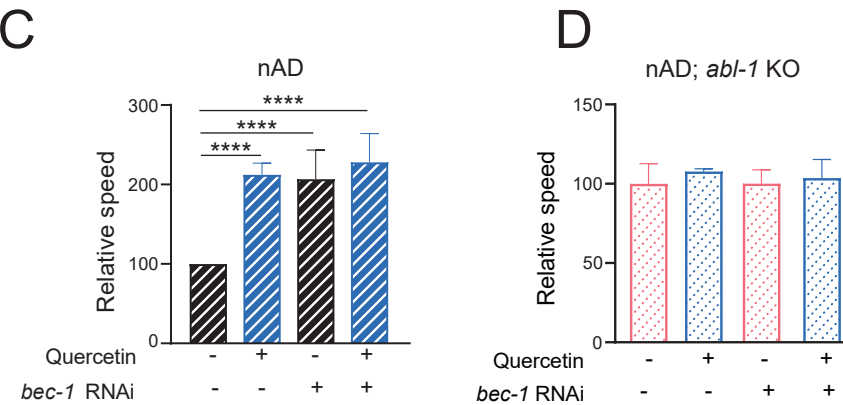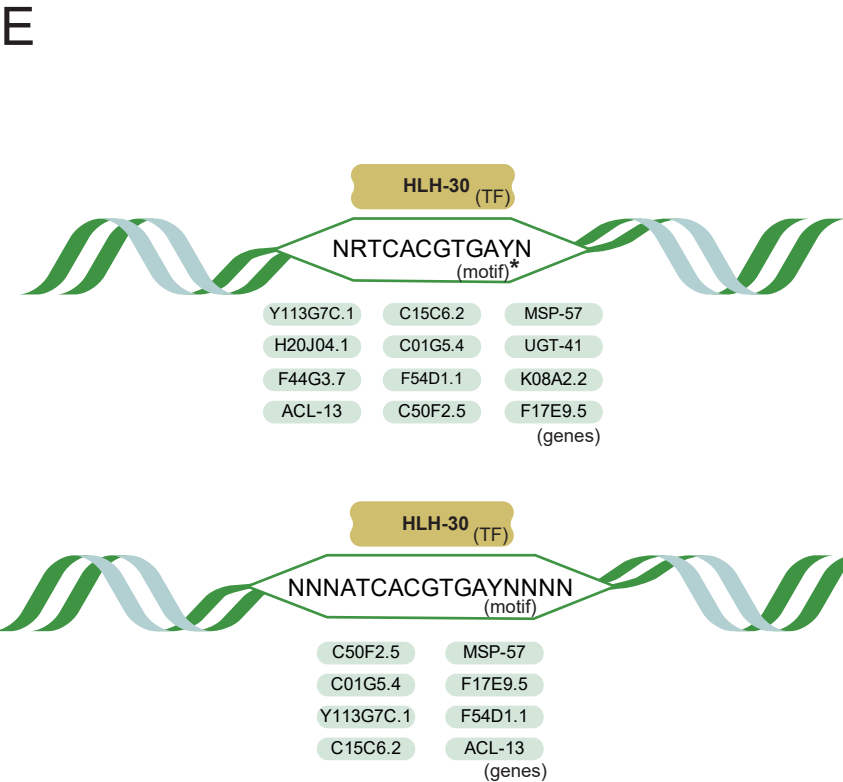

Supplement: Supplementary file 4 — Figure S4 [file 41420_2023_1592_MOESM4_ESM.pdf]
