# Supplementary material for: Abl depletion via autophagy mediates the beneficial effects of quercetin against Alzheimer pathology across species
Source: Cell Death Discov. 2023 Oct 14;9:376. doi: 10.1038/s41420-023-01592-x (PMC10576830; doi:10.1038/s41420-023-01592-x)
Supplement: Supplementary file 2 — Figure S2 [file 41420_2023_1592_MOESM2_ESM.pdf]

Figure S2

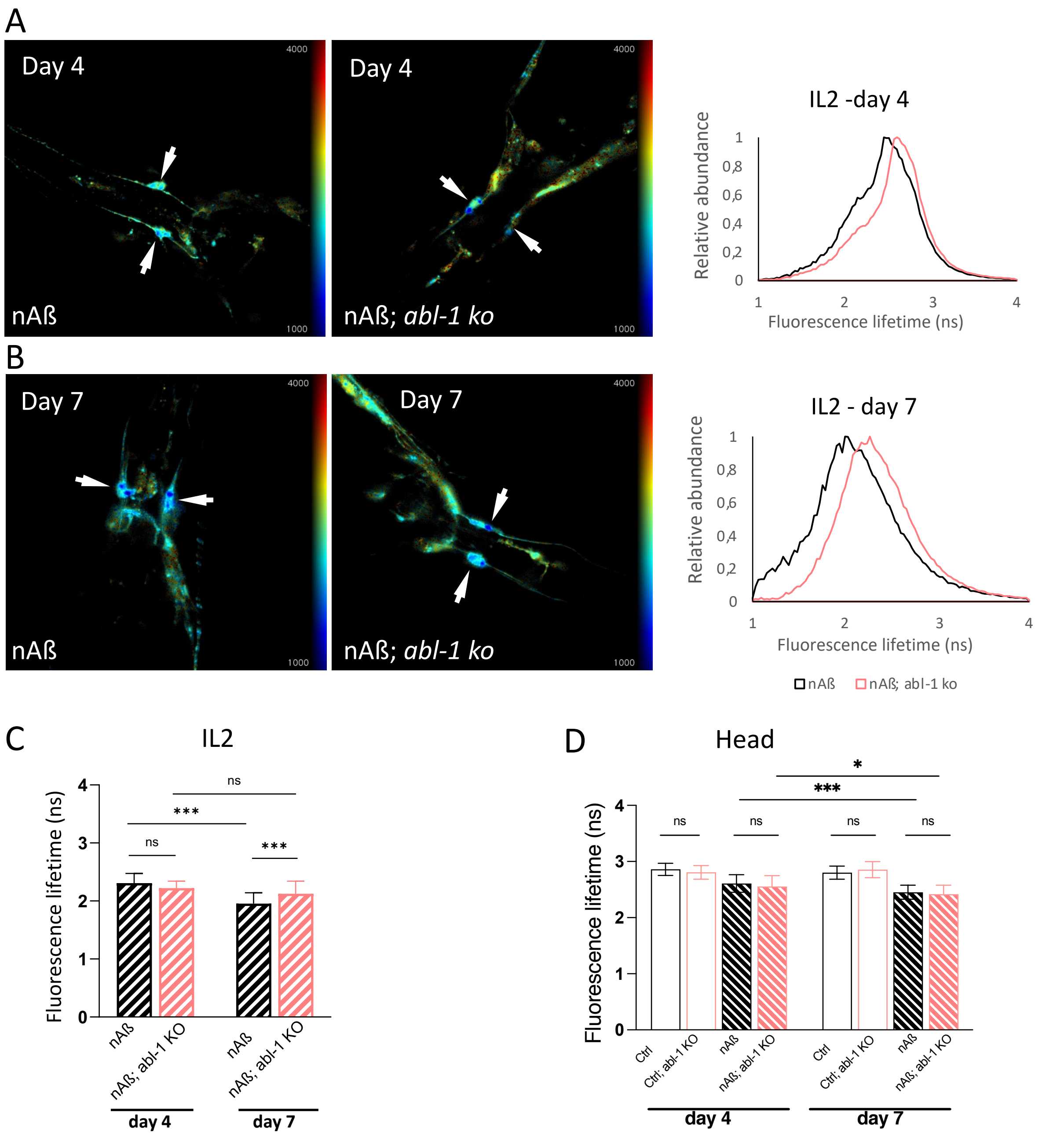

E **Table:** TCSPC-FLIM summary

Strain	Region	Age (day)	N	Fluorescence lifetime (ns)	Standard deviation	P vs nAß	P vs Ctrl	P vs Ctrl; abl-1 KO
**nAß**	Head	4	40	2,60	0,16	-	<0,0001	<0,0001
		7	40	2,45	0,13	-	<0,0001	<0,0001
	IL2	4	40	2,31	0,17	-	-	-
		7	40	2,07	0,22	-	-	-
**nAß; abl-1 KO**	Head	4	31	2,55	0,19	0,9767	<0,0001	<0,0001
		7	30	2,42	0,16	>0,9999	<0,0001	<0,0001
	IL2	4	31	2,22	0,12	0,1475	-	-
	IL2	7	30	2,15	0,22	0,0008	-	-
**Ctrl; abl-1 KO**	Head	4	29	2,81	0,12	<0,0001	0,9788	-
		7	28	2,85	0,14	<0,0001	0,9867	-
**Ctrl**	Head	4	40	2,86	0,11	<0,0001	-	0,9788
		7	40	2,80	0,12	<0,0001	-	0,9867
