# Supplementary material for: Abl depletion via autophagy mediates the beneficial effects of quercetin against Alzheimer pathology across species
Source: Cell Death Discov. 2023 Oct 14;9:376. doi: 10.1038/s41420-023-01592-x (PMC10576830; doi:10.1038/s41420-023-01592-x)
Supplement: Supplementary file 5 — Supplementary Figure Legends [file 41420_2023_1592_MOESM5_ESM.docx]

**Supplementary Figure Legends**

**Figure S1. A-F** Behavioral and physiological assay performed on neuronal Aβ-expressing (nAD) and control (wt) worms left untreated or treated with Lutein [100 µM], Lycopene [4.6 µM], or, Epigallocatechin gallate (EGCG) [0.64 µM]. **A** Body bends for minute in liquid media, error bars represent SEM (N=3, n=30), **** *P* < 0.0001 versus untreated condition, ###*P* < 0.001, ####*P* < 0.0001 versus wt. Statistical tests calculated using 2-way ANOVA (Tukey´s multiple comparisons test). **B** Percentage of 7 days old animals on the food after 2 hours from seeding them on the test plates. (N=3, n > 90). **P* <0.05, ***P* <0.01, *****P* < 0.0001 versus untreated condition, ###*P* < 0.001, #*P* < 0.05 versus wt. Statistical tests calculated using 2-way ANOVA (Tukey´s multiple comparisons test). **C,E** Kaplan-Meier survival curves of wild type (wt) **C** and neuronal Aβ-expressing (nAD) worms **E,** see **Table 2** for statistics**. D,F** Survival curves in response to heat shock performed on (wt) **D** and neuronal Aβ-expressing (nAD) **F** worms, see **Table 1** for statistics **G-H** Logarithmic fold change in base 2 (Log_2_ FC) of the average animals on food **G**, and of the mean lifespan **H,** with respect to the untreated conditions.

**Figure S2 A-E** Quantification of Aβ aggregation in neurons. **A-B** Representative TCPSC-FLIM images of day 4 (A) and day 7 (B) nematodes expressing wrmScarlet-Abeta(1−42) in neurons of wt (left panels) or *abl-1* mutant (Right panels) strains. The nematodes are false colored according to the fluorescence lifetime of mScarlet tagged Aβ with blue representing low lifetimes (= aggregated protein) and red indicating high lifetimes (= soluble protein). The white arrows indicate the IL2 neurons where Aβ aggregation starts. Histograms show pixel frequency distribution at determined lifetime in wrmScarlet-Abeta(1−42);wt (black lines) and wrmScarlet-Abeta(1−42); *abl-1 ko* (pink lines) animals. **C-D** Bar graphs show mean +/- SD Fluorescence lifetime for IL2 neurons (C) and for the head region (D). Two-way ANOVA followed by Tukey post-hoc test with 95% confidence interval was performed to assess statistical significance between strains and ages (N=3, n>10). **P* < 0.05; ***P* < 0.01; *****P* < 0.0001). **E** Table summarizing results and statistics from TCSPC - FLIM analysis.

**Figure S3. A-D** Behavioral and physiological assay performed on wild type (wt), neuronal Aβ-expressing (nAD) and *abl-1* KO crossed with nAD strain. **C** Survival curves in response to heat shock performed on worms treated with Imatinib (STI) [1 µM], see **Table 1** for statistics. **D** Body bends for minute in liquid media on worms left untreated or treated with STI [1 µM], error bar represent SD (N=3, n=30), *****P* < 0.0001. Statistical tests calculated using 2-way ANOVA (Tukey´s multiple comparisons test). **E** percentage of immobilized worms left untreated or treated with STI [1µM]. Immobilization was induced using Serotonin [10µM] in S-basal, see **Table** **4** for statistics. **F** Chemotaxis index of worms left untreated or treated with STI [1 µM]. Before to proceed with the chemotaxis assay, worms were starved for 2 hours either with (trained) or without (-) benzaldehyde [1%]. Bar graph represent mean ±SD (N=3, n>90), *****P* < 0.0001. Statistical tests calculated using 2-way ANOVA (Tukey´s multiple comparisons test).

**Figure S4. A** RT-qPCR of ABL mRNA in HEK293T-pIRES and HEK293T-APP^WT^ cells upon Quercetin [20 mM] treatment for 24 h. Actin was used as housekeeping gene. Statistical analyses performed by paired Student's t test (* p < 0.05; ** p < 0.001). **B** Normalized mRNA expression obtained from the RNA-Seq , using wild type and *abl-1* KO worms left untreated or treated with Quercetin [100 µM]. Values were normalized to wild type untreated. Bar graphs represent mean ± SD. *****P* < 0.0001 calculated by 2-way ANOVA (Tukey´s multiple comparisons test). **C-D** Relative speed of nAD and nAD; *abl-1* KO strains fed, starting from L4, HT115(DE3) bacteria transformed with either empty-vector or vector-expressing dsRNA against *bec-1* (*bec-1* RNAi), bar graphs shown mean ± SD of the normalized value to the untreated internal control nAD **C,** or to the untreated nAD;*abl-1* KO **D.** (N=3, n=95-177), *****P* < 0.0001 calculated by 2-way ANOVA (Tukey´s multiple comparisons test). **E** Schematic representation of the putative regulatory elements resulting from the analysis of the 171 DEGs. All genes listed on the scheme have at least one putative transcription factor (TF) binding sites for indicated motif; *indicates genes which contain at least 2 sites for the indicated motif. Motif´s nucleotide bases are presented in IUPAC code, N = any base, R= A or G, Y= G or T.
