# Supplementary material for: Abl depletion via autophagy mediates the beneficial effects of quercetin against Alzheimer pathology across species
Source: Cell Death Discov. 2023 Oct 14;9:376. doi: 10.1038/s41420-023-01592-x (PMC10576830; doi:10.1038/s41420-023-01592-x)

Supplemental Material
Original Blots

Red frames indicate the cropped blots used on the main figures

Supplemental Material to Figure 5C (original blots)

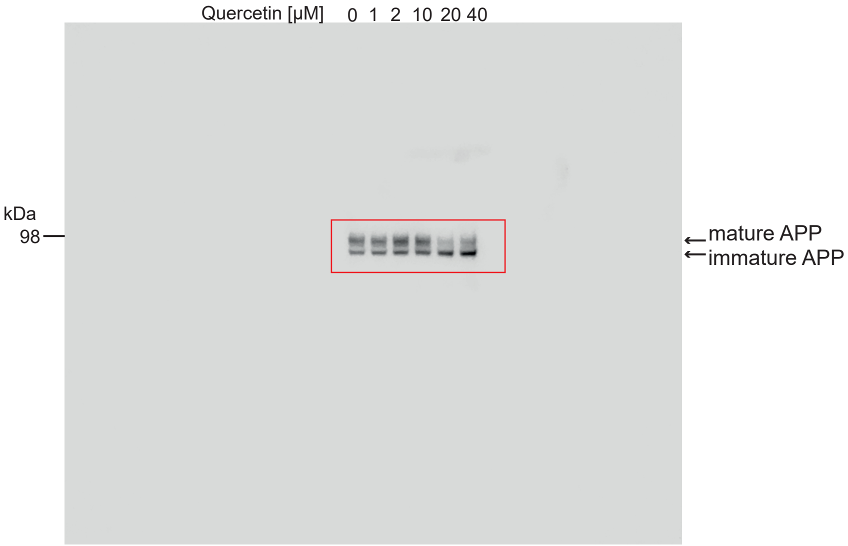

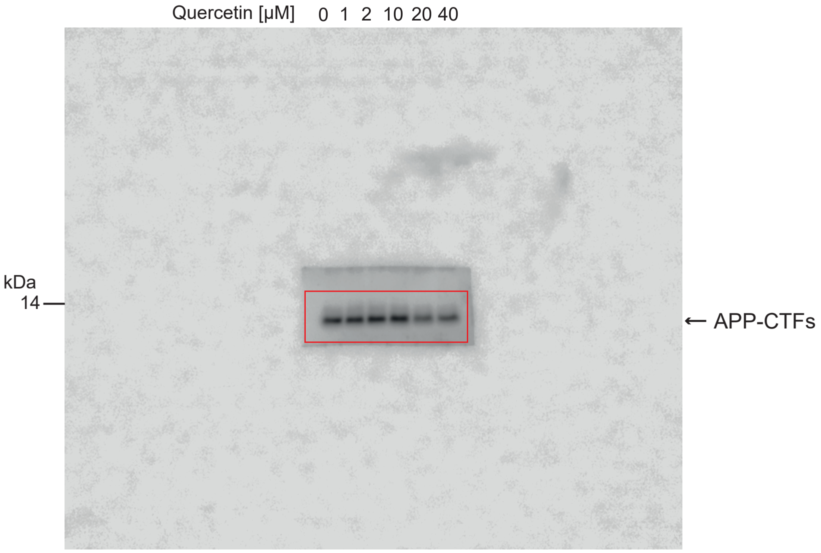

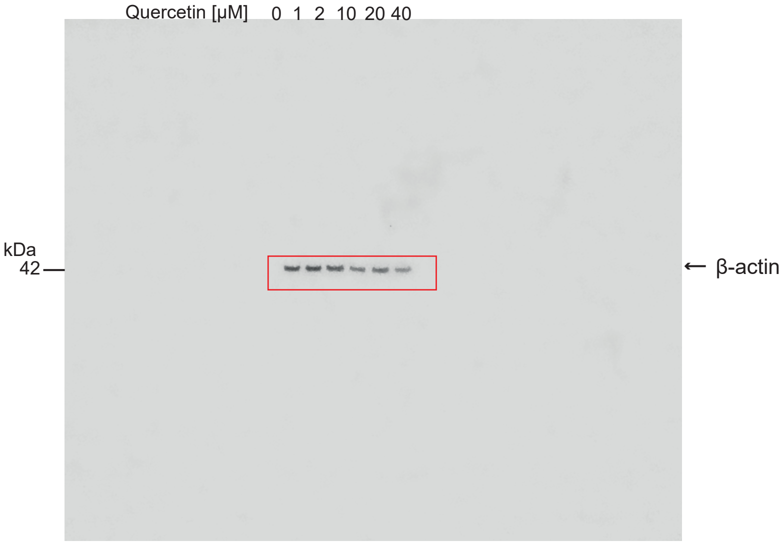

Supplemental Material to Figure 5D (original blots)

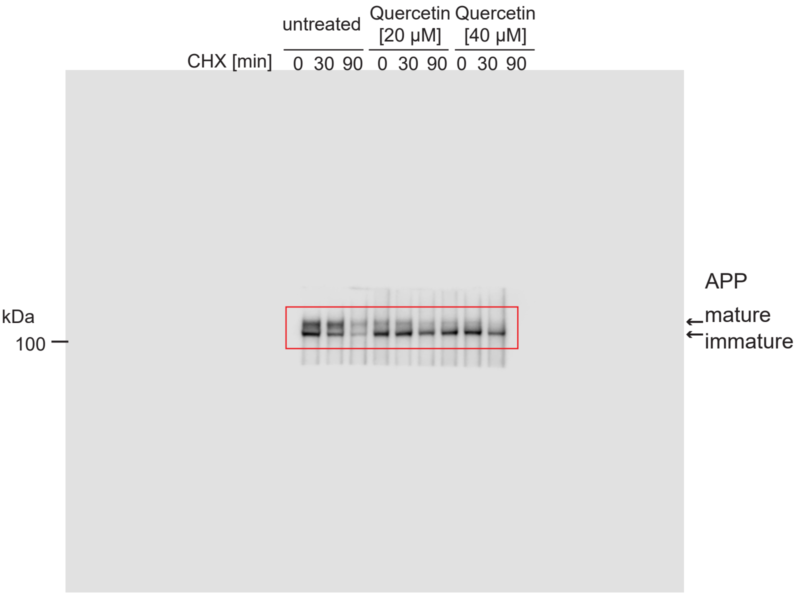

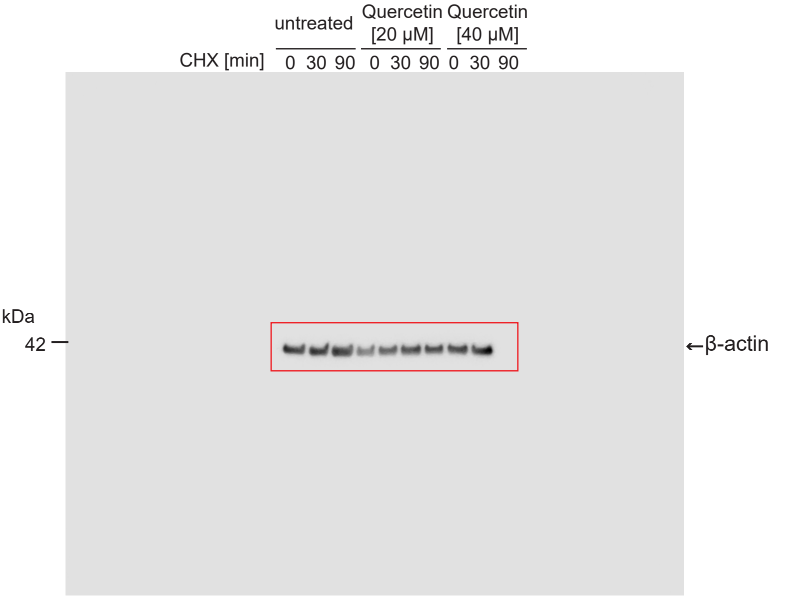

Supplemental Material to Figure 6A (original blots)

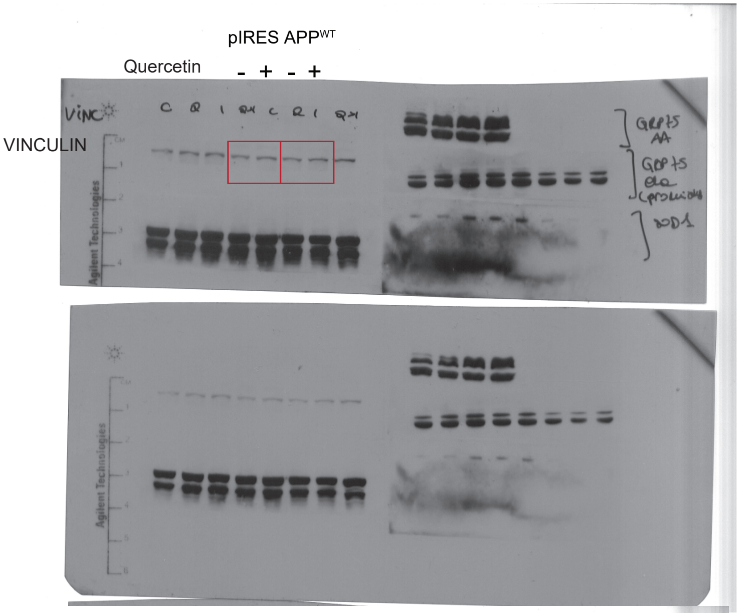

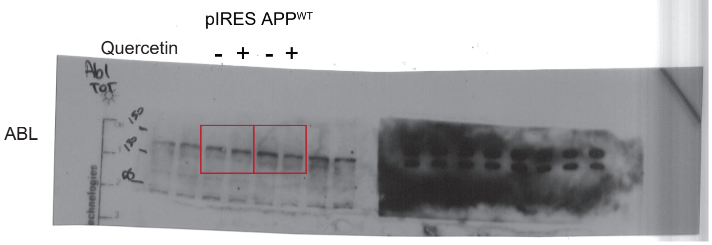

Supplemental Material to Figure 6B original blots)

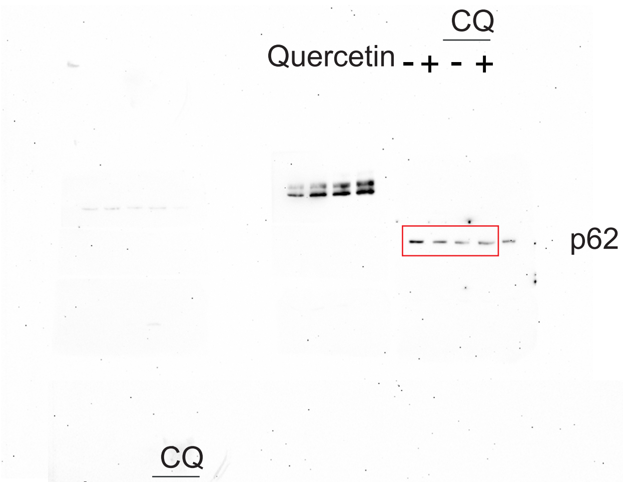

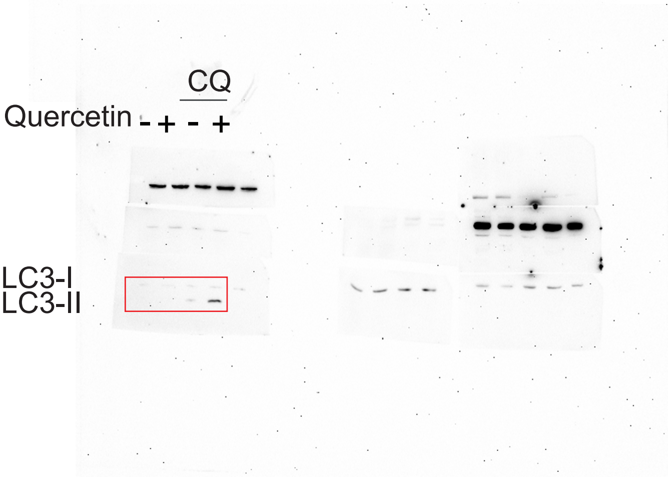

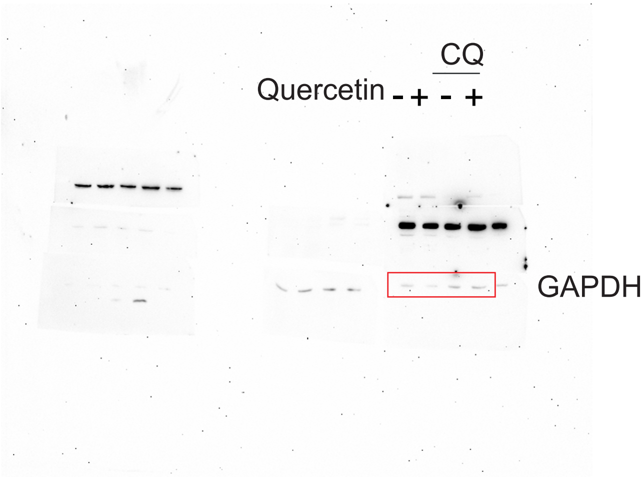

Supplemental Material to Figure 6C (original blots)

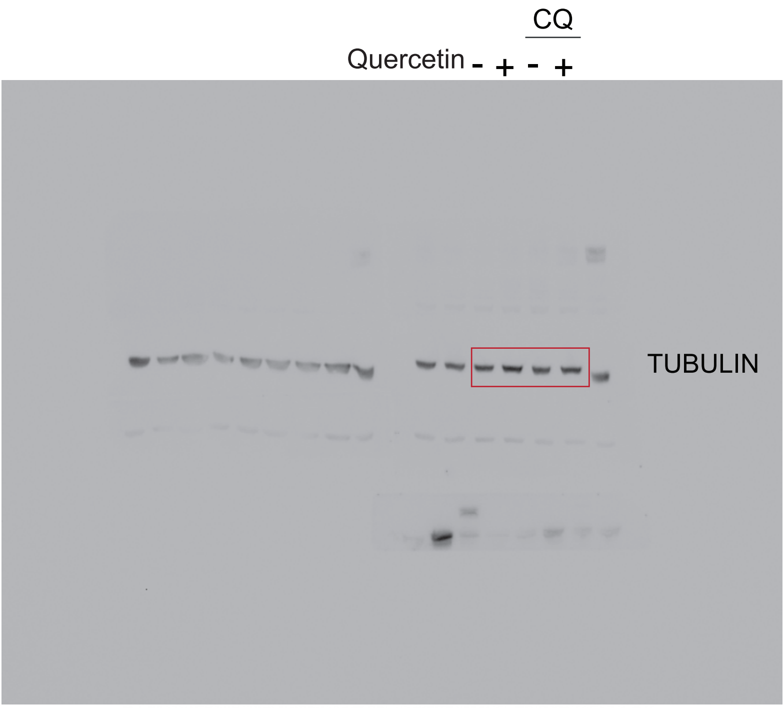

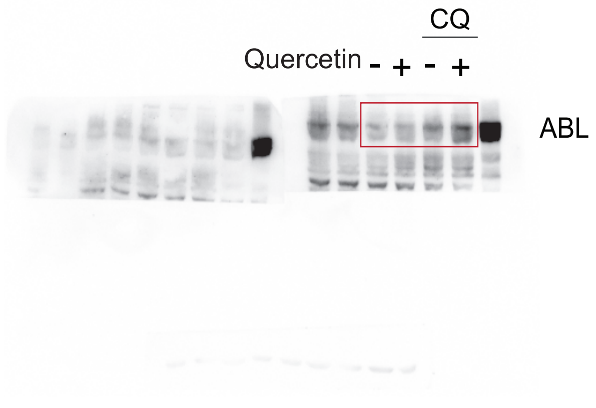

Supplement: Supplementary file 6 — Uncropped western blots [file 41420_2023_1592_MOESM6_ESM.pdf]
